# E183K Mutation in Chalcone Synthase C2 Causes Protein Aggregation and Maize Colorless

**DOI:** 10.3389/fpls.2021.679654

**Published:** 2021-06-23

**Authors:** Haixiao Dong, He Li, Yingjie Xue, Shengzhong Su, Shipeng Li, Xiaohui Shan, Hongkui Liu, Nan Jiang, Xuyang Wu, Zhiwu Zhang, Yaping Yuan

**Affiliations:** ^1^College of Plant Science, Jilin University, Changchun, China; ^2^Department of Crop and Soil Sciences, Washington State University, Pullman, WA, United States

**Keywords:** maize, colorless mutant, ethyl methyl sulfone, flavonoid biosynthesis, MutMap, prokaryotic expression, transient expression

## Abstract

Flavonoids give plants their rich colors and play roles in a number of physiological processes. In this study, we identified a novel colorless maize mutant showing reduced pigmentation throughout the whole life cycle by EMS mutagenesis. E183K mutation in maize chalcone synthase C2 (ZmC2) was mapped using MutMap strategy as the causal for colorless, which was further validated by transformation in Arabidopsis. We evaluated transcriptomic and metabolic changes in maize first sheaths caused by the mutation. The downstream biosynthesis was blocked while very few genes changed their expression pattern. ZmC2-E183 site is highly conserved in chalcone synthase among Plantae kingdom and within species’ different varieties. Through prokaryotic expression, transient expression in maize leaf protoplasts and stable expression in Arabidopsis, we observed that E183K and other mutations on E183 could cause almost complete protein aggregation of chalcone synthase. Our findings will benefit the characterization of flavonoid biosynthesis and contribute to the body of knowledge on protein aggregation in plants.

## Introduction

Flavonoids, which mainly consist of such compounds as chalcones, flavones, flavonols, anthocyanins, and proanthocyanins (Williams and Grayer, 2004), are a group of secondary metabolites that play important roles in multiple physiological processes. Anthocyanins and some other pigments are responsible for the red-blue color in flowers, seeds and other tissues, which could help plants attract pollinators or seed dispersers (Schaefer et al., 2004). Flavonoids have antioxidant functions by inhibiting the generation or reducing the levels of reactive oxygen species (ROS) (Agati et al., 2012); they are involved in UV protection (Schmelzer et al., 1988; Li et al., 1993; Bieza and Lois, 2001) and tolerance to other abiotic stresses (Li et al., 2017; Ren et al., 2019). Recent studies have observed associations between the biological activities of flavonoids and a variety of health benefits, including eyesight improvement (Ghosh and Konishi, 2007), cardiovascular health (Wallace et al., 2016), and cancer prevention (Lin et al., 2017).

Due to the success of isolating mutants with alterations in pigmentation (Koornneef, 1990; Holton and Cornish, 1995), the metabolites and key genes involved in flavonoid biosynthesis have been studied intensively in plants, including maize (Winkel-Shirley, 2001; Falcone Ferreyra et al., 2012; Eloy et al., 2017). In the upstream phenylpropanoid biosynthesis, phenylalanine is converted to 4-coumaroy-CoA by phenylalanine ammonia lyase (PAL), cinnamate 4-hydroxylase (C4H), and 4-coumarate CoA ligase (4CL). In the first two steps of flavonoid biosynthesis, 4-coumaroy-CoA is converted to naringenin chalcone by chalone synthase (CHS) and chalcone isomerase (CHI). Naringenin can be catalyzed into flavonols, anthocyanins, or flavones through different branches and enzymes. In addition to these structural genes, biosynthesis is controlled by transcriptional regulators, including R2R3 MYB, basic helix-loop-helix (bHLH) and WD repeat (WDR) proteins and their complexes (Jin et al., 2000; Gonzalez et al., 2008; Feller et al., 2011; Xu et al., 2015; Strygina et al., 2019; Sun et al., 2020). Considering the important functions of flavonoids and the biosynthesis as a colorful model for genetics and cell biology (Winkel-Shirley, 2001), it is inspired to discover novel genes and mutations affecting flavonoid biosynthesis.

Artificial mutants serve as essential materials for forward and reverse genetics. In Arabidopsis and rice, T-DNA insertion mutagenesis has been widely used (Krysan et al., 1999; Jeon et al., 2000). The success obtained with this model cannot be easily replicated in maize owing to the difficulty of large-scale transgene manipulation. However, other efficient methods, including irradiation, Mu transposon or chemical reagents, have been successfully implemented in maize (McCarty et al., 2005; Jia et al., 2016; Lu et al., 2018).

Traditional map-based cloning of mutants relies on repeatedly screening putative markers and evaluating the entire population, which is time-consuming and labor-intensive. Recently, a significant cost reduction was achieved by combining the bulked segregant analysis (BSA) strategy (Michelmore et al., 1991) with high-throughput sequencing. Individuals with extreme phenotypes are selected from each tail of the population and mixed together as a pool. Genotyping is only performed on chosen pools instead of the entire population. Distinct sequencing technologies (e.g., whole genome resequencing, RNA-seq, and exome capture sequencing) can be implemented, and different calculation methods were developed, such as the SNP-index (Abe et al., 2012), ED values (Hill et al., 2013) and G statistics (Magwene et al., 2011). Furthermore, population construction strategies evolved. Such methods as NGM (Austin et al., 2011), SHOREmap (Schneeberger et al., 2009), and BSR-seq (Liu et al., 2012) use an F2 or BC1 population derived from two lines with distant ecotypes. Abe et al. (2012) developed the MutMap strategy for chemical-induced mutation, in which the mutant line is crossed to the wild-type line with the same ecotype. These strategies have been implemented to accelerate the process of mutant mapping by direct genome sequencing in Arabidopsis (Cuperus et al., 2010; Austin et al., 2011), rice (Abe et al., 2012; Takagi et al., 2015), and maize (Lu et al., 2018; Tran et al., 2020).

In the process of gene expression, from DNA to a functional protein or RNA, modification or regulation frequently occurs and plays indispensable roles. Posttranslational regulatory steps include posttranslational modifications, protein degradation, and aggregation. Intracellular protein degradation is the breakdown of abnormal or superfluous proteins into smaller polypeptides or amino acids. Some misfolded proteins may escape from degradation, instead accumulating and clumping together to form aggregates (Tyedmers et al., 2010). One of the best-known examples of protein aggregation is sickle cell anemia (Clancy, 2008). Genetic mutation (E6V) occurs in the oxygen-carrying protein hemoglobin (HbS). Under low-oxygen conditions, HbS polymerizes and forms fibrous precipitates. As a consequence, the shape of red blood cells changes from a normal flexible and round shape to a rigid and sickle-like. Recently, the accumulation and aggregation of abnormal proteins was found to be a common feature of human neurodegenerative diseases, such as Alzheimer’s disease, Parkinson’s disease, Huntington’s disease, and amyotrophic lateral sclerosis (Boeynaems et al., 2018; Guo et al., 2018). Different from the brilliant picture in the animal field, studies about protein aggregation in plants remain rare. Although there are reports showing that stressful conditions could induce protein unfolding and aggregation to comprise cell survival (Nakajima and Suzuki, 2013; McLoughlin et al., 2019), there is little knowledge about how genetic mutations affect protein aggregation in plants, owing to the lack of related mutants.

In this study, combing ethyl methane sulfonate (EMS) mutagenesis and MutMap strategy, we identified and did gene mapping for a novel colorless maize mutant. The causal mutation proved to cause protein aggregation and dysfunction. Also, the effect of causal mutation on transcriptomic and metabolic levels were investigated.

## Materials and Methods

### Maize Growth and Sampling

For whole lifetime phenotypic observation, maize materials were grown in the field with regular management. In all other cases, materials were grown to three leaf stages in a greenhouse. For anthocyanin measurement, fresh tissues were used. For DNA, RNA, and metabolome extraction, samples were immediately frozen in liquid nitrogen and stored at −80°C.

### Anthocyanin Measurement

Anthocyanin was extracted with a methanol–HCl method (Lee and Wicker, 1991; An et al., 2017). Briefly, 0.1–0.2 g weighted tissues were grounded in liquid nitrogen and then soaked in 1 ml 1% HCl-methanol under darkness for 2 h with occasionally shaking for mixture. Optical density (OD) was measured using a UV spectrophotometer at 530, 620, and 650 nm. Anthocyanin concentration was calculated as [(OD530–OD620)−0.1 × (OD650–OD620)] × *v*/*m*, where *m* represents the weight of tissues (g), *v* is the volume (1 ml) of extraction buffer.

### Mutant Mapping

Mutant mapping was taken with a modified MutMap strategy (Abe et al., 2012). An F2 population was derived from the B73 and colorless mutant lines. The two parents and MP (pool of over 200 mutant-type F2 individuals) were subjected to whole genome sequencing. Library preparation and sequencing were performed by Biomaker (Beijing, China) using Illumina Hiseq4000 (PE150) following a standard protocol.

Bioinformatics analysis after sequencing was implemented following a commonly used approach. The raw fastq format files were filtered using NGSGCToolkit (Patel and Jain, 2012) to remove adaptors and low-quality reads. Clean reads were aligned to the B73_RefGen_v4 reference genome (Jiao et al., 2017) achieved from Ensembl-Plants^1^ using BWA (Li and Durbin, 2009). Picard^2^ was used to assess the alignment and mark PCR duplicates. GATK (McKenna et al., 2010) and samtools (Li et al., 2009) were used for variant calling. Finally, snpEff (Cingolani et al., 2012) was used for variant effect annotation.

Variants supported by more than two reads were treated as real variants. Homozygous variant was defined as one allele supported by at least four reads, while the other allele supported by no more than one reads, allowing the tolerance of sequencing errors. Variants that were homozygous and unique to the mutant line were used as markers for gene mapping. Variant-index was calculated as alternative allele frequency (read count for alternative allele/total read count) in mutant-type pool (MP). Lowess fitness was performed variant-index for smoothing using the “lowess” function in R (R Core Team, 2019).

### Sanger Sequencing Validation

For variant validation, the PCR products were directly sequenced using the corresponding PCR primers (Supplementary Table 7, primer #1). For plasmid construction, sequencing was implemented to confirm the accuracy of the construct using common primers in the vector. All sanger sequencings were performed by Sangon (Shanghai, China). 4peaks^3^ were used for the visualization of sequencing peaks. Sequence alignment was performed using pairwise sequence alignment tools available at EMBL-EBI^4^.

### Protein Structure Analysis

For protein 3D structure analysis, the ZmC2 protein sequence was submitted to swiss-model^5^ (Waterhouse et al., 2018). The chalcone synthase 1 (*Oryza sativa Japonica Group*) (NCBI accession: XP_015618054.1; PDB accession: 4yjy) with 92.15% alignment identity to ZmC2 was selected as a modeling template. Visualization of protein structure was performed using PyMOL (Schrodinger LLC, 2015).

### RNA Extraction, cDNA Synthesis, and Real-Time qRT-PCR

RNA was extracted from maize first sheaths or leaves using an Ultrapure RNA Kit (DNase I) (Order No. CW0597S, CWBIO, Beijing, China). cDNA was generated using UEIris II RT-PCR System for First-Strand cDNA Synthesis Kit (Order No. R2028, US Everbright, Suzhou, China). qRT-PCR was performed with three biological replicates using the Eco^TM^ Real-Time PCR system (Illumina) and 2x SYBR Green qPCR Master Mix (Order No. 522085, Bimake, Shanghai, China). The Real-Time qRT-PCR program had three steps: step 1 (Hot-Start DNA Polymerase Activation), 95°C for 30 s; step2 (PCR), 40 cycles of 95.0°C for 15 s and 60.0°C for 30 s; step 3 (Melt Curve), 95.0°C for 15 s, 60.0°C for 60 s, increment of 0.3°C increase/cycle to 95.0 and 95.0°C for 15 s. The primers for *ZmC2* [chalcone synthase C2 (*Zea mays*); Ensembl-Plants accession: Zm00001d052673] were CAGAAGGCGATCAAGGAGTG and GGTACATCATGAGGCGGTTC (product size: 151 bp) (Eloy et al., 2017). *ZmTUB2* [beta tubulin 4 (*Z. mays*); NCBI accession: NP_001105457] was used as internal control for normalization with the primers CTACCTCACGGCATCTGCTATGT and GTCACACACACTCGACTTCACG (product size: 297 bp) (Lin et al., 2014). The relative transcript levels were calculated using the 2^–ΔΔCT^ method (Livak and Schmittgen, 2001).

### RNA-seq

RNA-seq was performed on a pool of B73-type F2 progenies (two biological replicates, BP1 and BP2) and a pool of mutant-type F2 progenies (two biological replicates, MP1 and MP2). Each pool consists of 20 individuals’ first sheaths at the three-leaf stage. cDNA library preparation and sequencing were performed by Sangon (Shanghai, China) using Illumina HiseqXTen platform following a standard protocol. Bioinformatics analysis followed a common approach. Quality control was performed on raw reads using Trimmomatic (Bolger et al., 2014). Clean reads were further aligned to the reference genome B73_RefGen_v4 using Hisat2 (Kim et al., 2015). The R package “DESeq2” (Love et al., 2014) was used for differential expression analysis. Differentially expressed genes (DEGs) were defined as —log_2_FoldChange— ≥1 and *q*-value ≤ 0.05. Gene annotation was achieved from Ensembl-Plants BioMart^6^.

### Metabolite Analysis

Metabolic profiling was performed on a pool of B73-type F2 progenies (three biological replicates, BP1-3) and a pool of mutant-type F2 progenies (three biological replicates, MP1-3). Each pool consists of 50 individuals’ first sheaths at the three-leaf stage. Sample preparation and extraction, UPLC-MS/MS analysis was performed by Beijing Guoke Biotechnology (Beijing, China) following a widely targeted metabolomics method (Chen et al., 2013). In brief, samples were freeze-dried and crushed. A total of 100 mg powder was dissolved in 1.2 ml 70% methanol, followed by centrifugation and filtration. The sample extracts were analyzed using a UPLC-ESI-MS/MS system (UPLC, SHIMADZU Nexera X2^7^ ; MS, Applied Biosystems 4500 Q TRAP^8^). OPLS-DA was performed using R package “MetaboAnalystR” (Chong and Xia, 2018). Significantly regulated metabolites between groups were determined by VIP (Variable Importance in Projection) ≥1 and | Log2FoldChange| ≥ 1. Identified genes and metabolites were mapped to KEGG pathway database^9^ (Kanehisa and Goto, 2000) and visualized using the R package “pathview” (Luo and Brouwer, 2013).

### Protein Conservation Analysis

For conservation analysis among species, ZmC2 was blast against Reference proteins (refseq_protein) database in NCBI^10^ and 1,582 proteins with 358--405 amino acid lengths were selected. Multiple sequence alignment (MSA) was performed using Clustal-Omega^11^ (Madeira et al., 2019). The MSA results were further analyzed and visualized using Mview^12^. Phylogenetic tree was visualized using Evolview v2 (He et al., 2016). For conservation analysis within species, variants information of ZmC2, ZmWHP1, OsCHS1, and AtCHS were achieved from Ensembl-Plants^13^.

The expression pattern for ZmC2 and ZmWHP1 in 23 tissues spanning multiple stages of maize development (Walley et al., 2016) were achieved from Expression Atlas^14^ (Papatheodorou et al., 2020).

### Gene Cloning and Plasmids Construction

A longer precursor containing the full CDS of *CHS* (*ZmC2* or *ZmWHP1*) was cloned using cDNA as template (Supplementary Table 7, primer #4, #5). Point mutations at E183 site including E183K, E183R, and E183D were induced using specific primers containing designed mutations (Supplementary Table 7, primer #6–8) following the overlap PCR strategy (Ho et al., 1989). For prokaryotic expression, the CDS was cloned into the pPH vector (preserved in our lab) using the double-digest system (*Nde*I and *Xho*I restriction enzymes) to generate the fusion protein His6-tag:CHS (Supplementary Table 7, primer #9, #10). The R12E point mutation and A2_P20 deletion mutation (A2_P20del) were induced using specific primers (Supplementary Table 7, primer #11, #12). For transient expression in maize leaf protoplasts, CDS was cloned into the pSATN1-GW vector using the Gateway Cloning System (Invitrogen) to generate the CHS:GFP-tag fusion protein. For transformation into *Arabidopsis thaliana*, CDS was cloned into pEarleyGate101 vector using the Gateway system to express CHS:YFP-tag fusion protein (Supplementary Table 7, primer #13–15).

### Prokaryotic Expression, Protein Purification, and Western Blot

For prokaryotic expression, the constructed plasmid was transformed into JM109 *Escherichia coli* cells. Prokaryotic expression was induced for 16-20 h at 18°C after adding IPTG. Cytolysis was performed using repeated freeze-thaw and lysozyme. The soluble His-tag fusion proteins were purified using a Ni-NTA column.

Western blotting was performed using a Western Blot Kit (Mouse) with a PVDF membrane (Order No. C600393, Sangon, Shanghai, China) following the instructions of the manufacturer. Proteins were transferred from gel to PVDF membrane using a semidry method. The first antibody used was an anti-His mouse monoclonal antibody, and the second antibody used was HRP-conjugated goat anti-mouse IgG. ECL luminescence reagent was used as the substrate of HRP, and X-film was used in the darkroom to capture the luminescence.

### Maize Leaf Protoplasts Extraction, Plasmids Transformation, and GFP Observation

Maize protoplast isolation and transformation were carried out as previously described in *A. thaliana* (Zhai et al., 2009) with some modifications. In brief, B73 was cultivated under darkness for approximately 10 days until the two-leaf stage. The second leaves were sliced vertical to the leaf vein into 1-mm thicknesses and used for protoplast extraction. Plasmid DNA was transformed into protoplasts using the PEG-Ca^2+^ method. Between 12 and 24 h after transformation, protoplasts were scanned for GFP signals using an inverted fluorescence microscope (Nikon, Japan).

### Arabidopsis thaliana Transformation and YFP Observation

Constructed plasmids were transformed into *Agrobacterium tumefaciens* strain GV3101. Transformation of Arabidopsis was done following the vacuum infiltration method (Bechtold et al., 1993). Transformed T1 seedlings were selected with 0.1% glufosinate ammonium (Basta), followed by PCR and RT-PCR detection (Supplementary Table 7, primer#2). *AtACT2* [actin 2 (*A. thaliana*); TAIR accession At3g18780] was used as internal control (primer #3). Leaves of positive lines were subjected to protoplasts extraction and YFP observation following a similar procedure as “maize leaf protoplasts extraction and GFP observation.”

## Results

### Phenotypic Observation of a Colorless Maize Mutant

**FIGURE 1:**
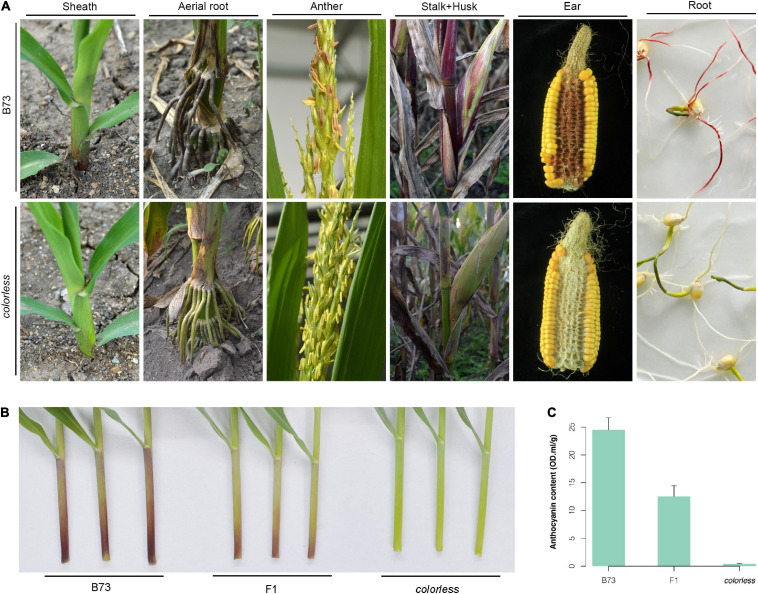
Phenotypic observation of a colorless mutant.**(A)** Whole lifetime comparison of B73 and mutant. Sheath was observed at seedling stage; aerial root and anther, flowering stage; stalk and husk, late milk stage; ear, after harvest; root, germination of seeds under light. **(B,C)** Observation and anthocyanin content measurement of the first sheath of B73, mutant and their hybrid. Seedlings were grown in greenhouse to three-leaf stage. Error bars indicate standard deviation (SD) of over three biological replicates.

A novel colorless mutant was screened from an EMS-induced mutant library for the maize B73 inbred line. Throughout the life cycle, this mutant showed reduced level of pigmentation, especially in sheath, aerial roots, cobs, etc., which are tissues expected to contain notably abundant pigment (Figure 1A).

The mutant was crossed with wild-type B73. Compared to B73 with red/purple leaf sheaths and high anthocyanin content, the mutant line showed green leaf sheaths with almost zero anthocyanin content, and the F1 hybrids showed intermediate color type and anthocyanin content (Figures 1B,C). Investigation of sheath color in the F2 population showed a 3:1 segregation ratio of colored individuals to colorless individuals. All these results indicate that the colorless phenotype is controlled by a single recessive allele.

### Mutant Mapping

**FIGURE 2:**
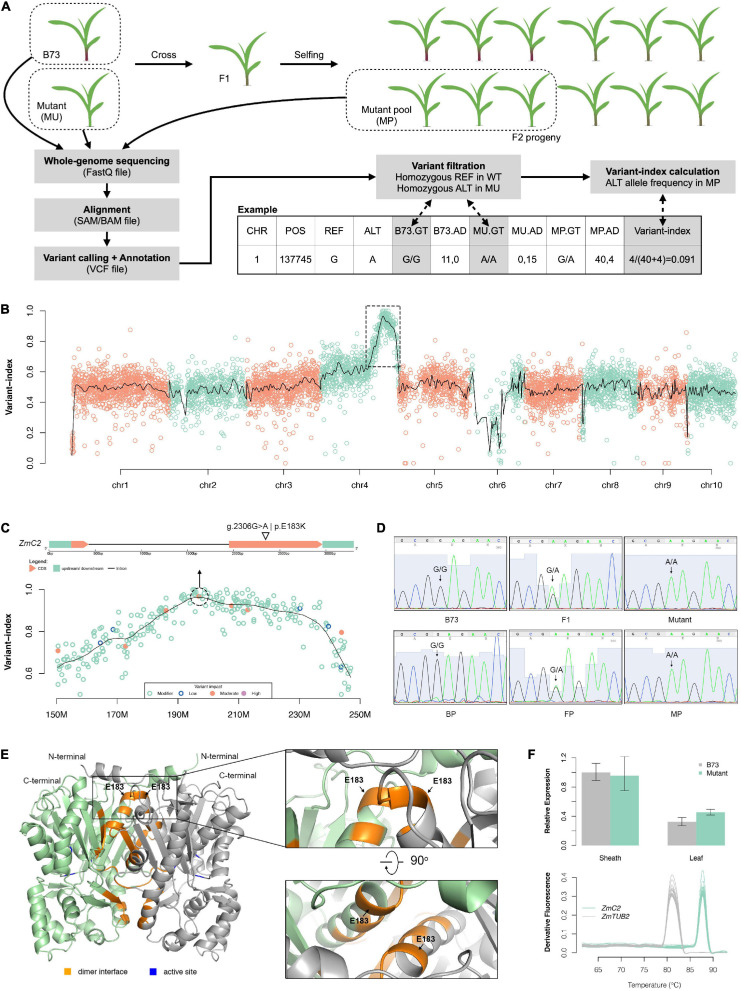
Mapping of the colorless mutant. **(A)** Schematic diagram of the mutant mapping strategy. **(B)** Genome-wise visualization of variant-index. The black curve represents smoothing values by loess regression. The dashed-line box circles out the candidate region (chr4:150M–250M) harboring causal mutation. **(C)** Close-up visualization of variant-index in chr4: 150M–250M region (bottom) and gene structure of *ZmC2* (top). **(D)** Sanger sequencing validation on g.2306G > A mutation of *ZmC2* in parental, F1 and F2 progeny. BP, pool of B73-type F2 progeny; FP, pool of F1-type F2 progeny; MP, pool of mutant-type F2 progeny. **(E)** Three-dimensional (3D) structure of ZmC2. OsCHS1, chalcone synthase 1 (*Oryza sativa Japonica Group*) (GenBank: XP_015618054.1; PDB: 4yjy) with 92.15% alignment identity to ZmC2 serves as modeling template. **(F)** Expression pattern of *ZmC2* in B73 and *colorless* mutant. The top panel displays qRT-PCR results and bottom panel displays melt-curves. *ZmTUB2*, beta tubulin 4 (*Zea mays*) (NCBI accession: NP_001105457) serves as internal control. The first sheath and first leaf at three-leaf stage were used here.

To identify the causal mutation, we used MutMap pipeline and an F2 population derived from B73 and the colorless mutant (Figure 2A). Over 200 F2 offspring showing mutant-type phenotype were mixed together as a MP. Whole genome sequencing was implemented on two parents and MP, generating approximately 232 million clean reads for B73, 333 million clean reads for the mutant, and 1,180 million clean reads for MP (Supplementary Table 1). Over 95% of these reads were mapped to maize reference genome (B73_RefGen_v4), and the average depth for the two parents was 14.2x and 19.4x, while 61.7x for MP. Cov_ratio_5x (position covered by at least five reads) for three samples were all over 96%. The high coverage and depth make it reliable for the detection of variants and calculation of allele frequencies.

In total, 176,899 variants (137,925 SNPs and 38,974 indels) were identified for the three samples (Supplementary Figure 1A). Over 90% of the variants were shared by all three samples and were treated as natural variants during cultivar and multiplication. After comparing the two parents, 5,654 variants (5,364 SNPs and 290 indels) that were unique and homozygous in the mutant were filtered and further used as markers for gene mapping. Of all these variants, there were 2,730 G- > A and 2,465 C- > T substitutions, together occupying 91.9% (Supplementary Figure 1B).

Variant-index was calculated as the allele frequency of the alternative alleles in MP for these markers. Subsequent smoothing showed that there is only one peak across the whole genome and located at Chr4:150 M-250 M (Figure 2B). Summit of the peak is Chr4:195678521. In the close up look of this candidate region, there is a G > A mutation with missense effect at Chr4:196895508, corresponding to g.2306G > A | p.E183K mutation in gene Zm00001d052673 (chalcone synthase C2, *ZmC2*) (Figure 2C and Supplementary Table 2). To validate this mutant site, the surrounding region was cloned and subjected to Sanger sequencing. The Sanger sequencing results were consistent with the results of the whole genome sequencing, as the genotype for B73 or B73-type F2 progeny is G/G, that of the mutant or mutant-type F2 progeny is A/A, that of F1 or F1-type F2 progeny is G/A (Figure 2D).

*ZmC2* is known to encode a naringenin-chalcone synthase (ZmC2, or ZmCHS2; NCBI accession: NP_001142246.1; Ensembl-Plants accession: Zm00001d052673_P001); the enzyme catalyzes the reaction 4-coumaroyl-CoA + malonyl-CoA → naringenin chalcone, which is the first dedicated step in flavonoid biosynthesis. Three-dimensional (3D) protein structure was built for ZmC2 based on OsCHS1 (chalcone synthase 1 (*Oryza sativa Japonica group*); NCBI accession: XP_015618054.1) (Supplementary Figure 2). ZmC2 is considered to exist as a homo-dimer and the E183 site is located on the dimer interface (Figure 2E). Furthermore, qRT-PCR showed that E183K mutation has no effect on the expression of *ZmC2* in the sheath or leaf (Figure 2F).

### Transgene Validation of ZmC2-WT and ZmC2-E183K in Arabidopsis

**FIGURE 3:**
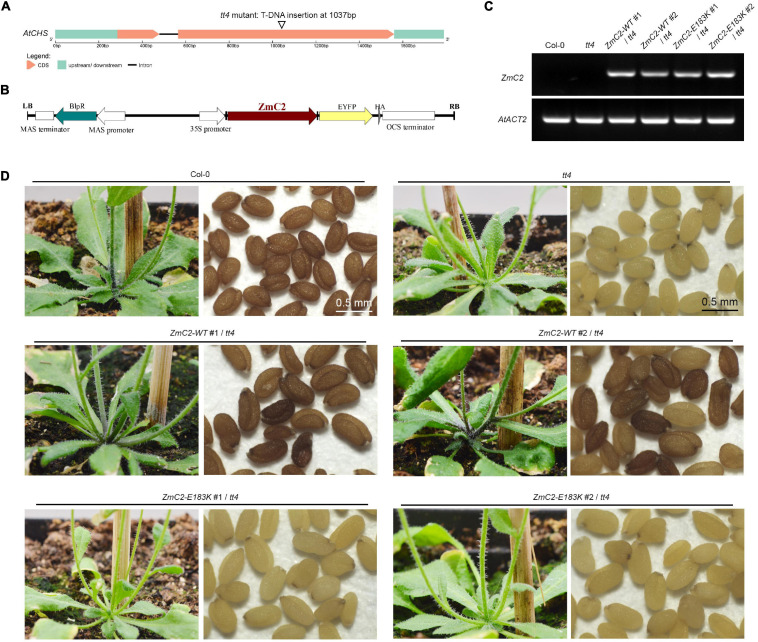
Transformation of ZmC2-WT and ZmC2-E183K in *Arabidopsis thaliana tt4* mutant. **(A)** Structure of Arabidopsis chalcone synthase (*AtCHS*, At5g13930) and the *tt4* mutant. **(B)** Schematic diagram of transgene construct from T-DNA leaf border (LB) to right border (RB). **(C)** RT-PCR detection of *ZmC2* in Arabidopsis. *AtACT2* [actin 2 (*Arabidopsis thaliana*), At3g18780] was used as internal control. **(D)** Phenotypic comparison. Two independent transgenic lines were displayed (#1 and #2).

There is a single homolog for ZmC2 in *A. thaliana*, that is At5g13930 (Chalcone and stilbene synthase family protein, *AtCHS*), and the alignment identity for them is 82.7% (Supplementary Figure 2). The available transparent testa 4 (*tt4*) mutant contains a T-DNA insertion in the second exon of *AtCHS* (Figure 3A). To explore the effect of E183K mutation on the activity of ZmC2. The two types of ZmC2, wild-type (-WT) and mutant-type (-E183K) were transformed into *tt4* mutant separately (Figure 3B). For each allele, more than two independent lines were gained (Figures 3C,D). In comparison, Col-0 could accumulate strong red pigmentation in its stem and seeds, while *tt4* mutant shows no pigmentation in these tissues. Transformation with ZmC2-WT could recover the mutant phenotype to distinct levels, while ZmC2-E183K cannot. These findings indicate that the E183K mutation in ZmC2 leads to its dysfunction and maize colorless.

### Transcriptomic and Metabolic Changes Caused by ZmC2-E183K Mutation in Maize Sheath

To investigate the effect of ZmC2-E183K on flavonoid biosynthesis and other related mechanisms, RNA-seq (two biological replicates) and widely targeted metabolome (three biological replicates) were performed on the first sheaths of B73-type F2 progeny (BP) and those of mutant-type F2 progeny (MP).

**FIGURE 4:**
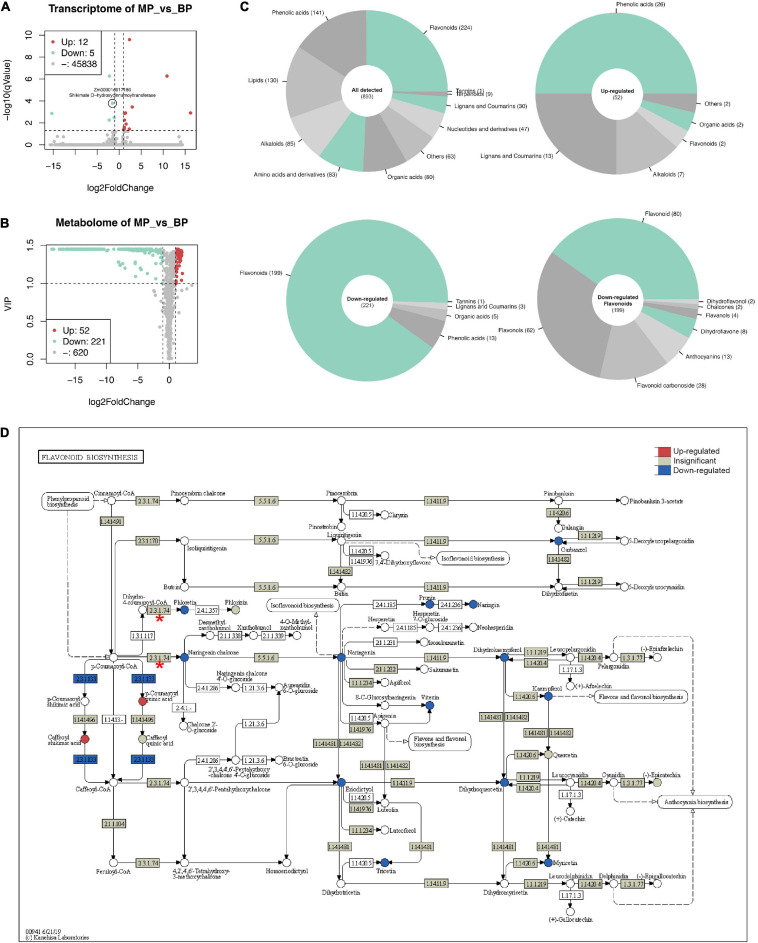
Transcriptomic and metabolic changes in maize first sheath due to ZmC2-E183K mutation. RNA-seq and widely targeted metabolome were performed on the first sheaths of B73-type F2 progeny (BP) and those of mutant-type F2 progeny (MP). Seedlings were grown to three-leaf stage in a greenhouse. For each type of sample, multiple individuals were pooled together. RNA-seq was performed with two biological replicates, and metabolome with three biological replicates. **(A)** Volcano plot for RNA-seq. The cut-off for differentially expressed genes (DEGs) is | log2FoldChange| ≥ 1 and *q*-value ≤ 0.05. Zm00001d017186 (Shikimate O-hydroxycinnamoyltransferase) is labeled out as the only DEG related to flavonoid biosynthesis. **(B)** Volcano plot for metabolome results. The cut-off for differentially expressed metabolites is | log2FoldChange| ≥ 1 and VIP ≥ 1. **(C)** Classification of metabolites. Upper left, all detected metabolites; upper right, up-regulated metabolites; lower left, down-regulated metabolites; lower right, more detailed classification of down-regulated flavonoids. **(D)** View of gene expression and metabolite changes in flavonoid biosynthesis (kegg-zma00941). In the pathway, circle represents metabolites and box represents reactions and genes. 2.3.1.74 with red asterisk is *ZmC2* involved reaction; 2.3.1.133 in blue color is Zm00001d017186 involved reaction.

RNA-seq generated 26.4–31.2 million pairs of reads for each sample, over 80% of the reads could be mapped to the reference genome in proper pairs (Supplementary Table 3). A total of 45,855 genes were detected in at least one sample. Seventeen genes were determined as DEGs (cut-off: | log2FoldChange| ≥ 1 and *q*-value ≤ 0.05), where 12 genes were up-regulated, and 5 were down-regulated. Zm00001d017186 (Shikimate O-hydroxycinnamoyltransferase) is the only DEG with slight down-regulation related to flavonoid biosynthesis (Figure 4A).

A total of 893 metabolites were identified and quantified in the widely targeted metabolome (Figure 4B). Of them, 52 were up-regulated while 221 were down-regulated in MP (cut-off: | log2FoldChange| ≥ 1 and VIP ≥ 1). The up-regulated metabolites mainly consist of phenolic acids (26), lignans and coumarins (13), while down-regulated metabolites mainly consist of flavonoids (199), and phenolic acids (13). The 199 down-regulated flavonoids could be further classified into flavonoid (80), flavonols (62), flavonoid carbonoside (28), and anthocyanins (13) (Figure 4C).

In flavonoid biosynthesis (kegg-zma00941), it is obvious that most of the detected metabolites in the downstream of ZmC2 involved reaction (EC: 2.3.1.74) showed significant decrease (Figure 4D). Also, detected metabolites in the downstream pathways, including anthocyanin biosynthesis (kegg-zma00942) and flavone and flavonol biosynthesis (keg-zma00944) showed significant decrease (Supplementary Figures 3, 4). In phenylpropanoid biosynthesis, the upstream of flavonoid biosynthesis, several phenolic acids were up-regulated. Of them, p-coumaric acid, caffeic acid, ferulic acid, and scopoletin are in the same path to generate scopolin, indicating this may be the major shunt (Supplementary Figure 5). In summary, ZmC2-E183K mutation caused blocking of downstream pathways and the shunt of metabolites in the upstream pathways without affecting gene expression.

### Conservation Analysis of Chalcone Synthase C2 and the E183 Site

**FIGURE 5:**
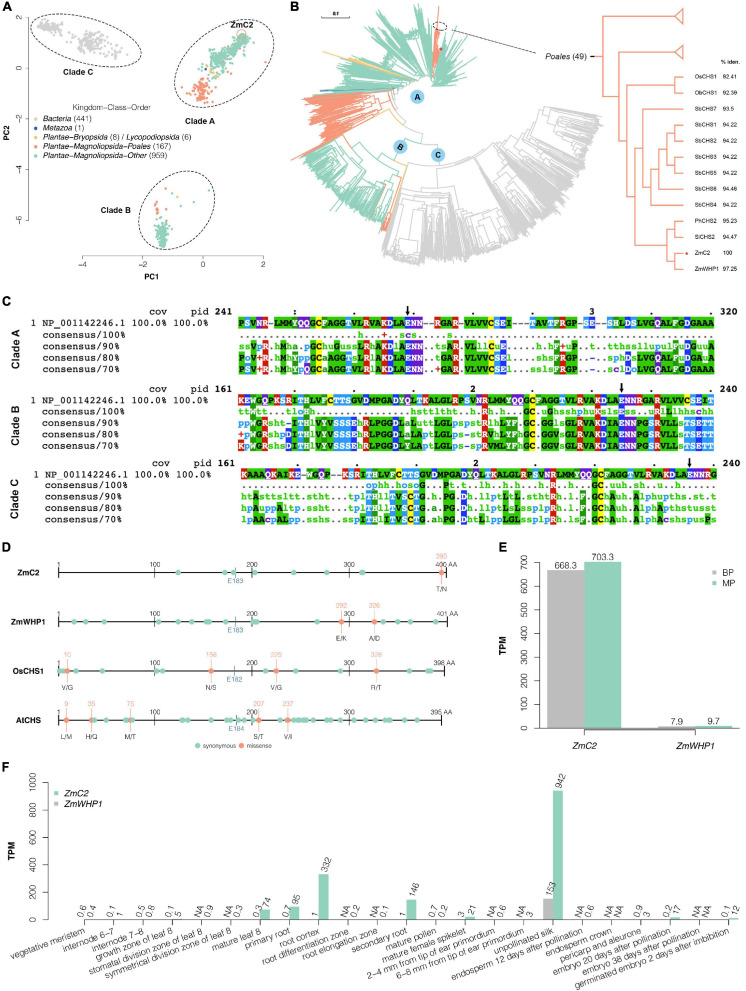
Conservation analysis of ZmC2 and E183 site. **(A)** Principal component analysis after multiple alignment of ZmC2 and its 1581 homologs. The dots are colored based on Kingdom-Class-Order of these proteins’ species. **(B)** Phylogenetic tree of ZmC2 and its homologs. The branch colors are consistent with dot colors in **(A)**. The right panel is a pruned view of 49 proteins belonging to Poales (class) containing ZmC2. OsCHS1, chalcone synthase 1 (*Oryza sativa Japonica Group*) (NCBI accession: XP_015618054.1); ObCHS1, PREDICTED: chalcone synthase 1 (*Oryza brachyantha*) (NCBI accession: XP_006662967.1); SbCHS1-7, chalcone synthase 1-7 (*Sorghum bicolor*) (NCBI accession: XP_002450874.1, , , XP_002450870.1, XP_002449616.1, XP_002450877.1, and XP_002450876.1); PhCHS2, chalcone synthase C2 (*Panicum hallii*) (NCBI accession: XP_025827694.1); SiCHS2, chalcone synthase C2 (*Setaria italica*) (NCBI accession: XP_004979391.1); ZmWHP1, chalcone synthase WHP1 (*Zea mays*) (NCBI accession: NP_001149022.1). % iden. means the percentage of identity of the protein and ZmC2. **(C)** Mview for multiple alignment of ZmC2 (NCBI accession: NP_001142246.1) and its homologs within each clade. The black arrow labels the corresponding site for ZmC2-E183. **(D)** Within species’ variations for ZmC2 (Ensembl-Plants accession: Zm00001d052673_T001), ZmWHP1 (Ensembl-Plants accession: Zm00001d007403_T001), OsCHS1 (Ensembl-Plants accession: Os11t0530600-01), and AtCHS (Ensembl-Plants accession: AT5G13930.1). This variation data sets consists of multiple varieties or cultivars and are available at Ensembl-Plants (http://plants.ensembl.org/index.html). The blue line and text labels the corresponding site for ZmC2-E183. **(E)** RNA-seq results of *ZmC2* and *ZmWHP1* in the first sheath of B73-type F2 progeny (BP) and mutant-type F2 progeny (MP). **(F)** Expression pattern of *ZmC2* and *ZmWHP1* in 23 maize tissues (Walley et al., 2016). The expression information is achieved from Expression Atlas (https://www.ebi.ac.uk/gxa/home; Papatheodorou et al., 2020). NA means data not available.

Multiple alignment was performed on ZmC2 and its 1581 homologs. Of them, 441 proteins belong to Bacteria (Kingdom), 1 belongs to Metazoa (Kingdom), 167 belong to Plantae (Kingdom)-Magnoliopsida (Class)-Poales (Order), and the rest belong to the other orders or classes in Plantae (Kingdom). Principal components analysis (Figure 5A) and phylogenetic analysis (Figure 5B) showed that these proteins could be grouped into three clades (A, B, and C). Multiple alignment was further performed on ZmC2 and proteins within each clade (Figure 5C). Proteins in clade C were all from Bacteria (Kingdom) and very different from ZmC2 at E183 site and surrounding region, while proteins from Plantae (Kingdom) were all in clade A/B and they were highly conserved at ZmC2-E183 site and surrounding region. ZmC2 is in a cluster of 49 proteins belonging to Poales (Order) (Figure 5B). The most similar protein for ZmC2 was ZmWHP1 [chalcone synthase WHP1 (*Z. mays*); NCBI accession: NP_001149022.1] with 97.25% identity. These two maize chalcone synthase (CHS) was in the same branch with CHS from *Setaria italica*, *Panicum hallii*, and seven CHS from *Sorghum bicolor*. Furthermore, we explored the conservation of chalcone synthase within species but among different varieties. Publicly available variation information was used for ZmC2, ZmWHP1, OsCHS1, and AtCHS (Figure 5D). There were few missense variations exist in these proteins, and none of them was at ZmC2-E183 site. These findings suggested that the corresponding ZmC2-E183 site in chalcone synthase have been kept conserved during evolution in Plantae (Kingdom) and during cultivar or domestication in different varieties within species.

Given that the highly identical ZmWHP1 cannot complement the dysfunction of ZmC2, we explored the expression pattern of both genes. From the RNA-seq results on the first sheaths of MP_vs_BP, we found that *ZmC2* had much higher (about 100 times) transcription level than *ZmWHP1*. Besides, ZmC2-E183K mutation did not cause a differential regulation of *ZmWHP1* (Figure 5E). Moreover, we used a publicly available RNA-seq data on 23 tissues spanning the vegetable and reproductive stages of maize development (Walley et al., 2016). In most tissues, the expression of *ZmC2* was much higher than *ZmWHP1* (Figure 5F). All these findings showed that *ZmC2* and *ZmWHP1* are independently regulated, and *ZmC2* plays the major role in flavonoid biosynthesis.

### E183K Mutation Causes Protein Aggregation of ZmC2

**FIGURE 6:**
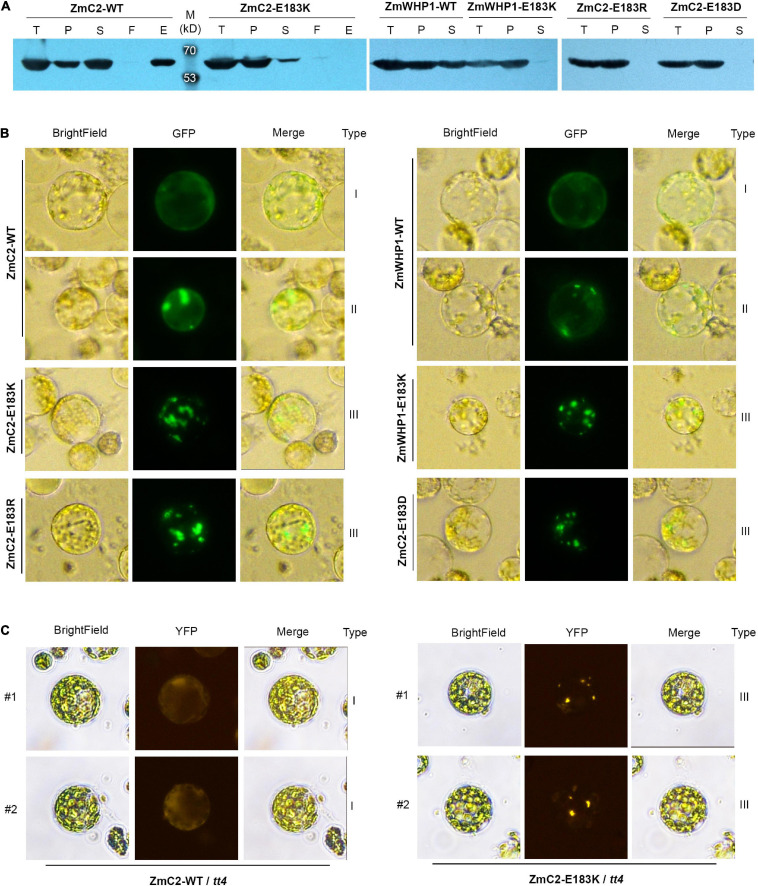
Mutation on E183 causes protein aggregation of chalcone synthase (CHS). ZmC2/ZmWHP1-WT represents the wild-type chalcone synthase ZmC2/ZmWHP1; ZmC2/ZmWHP1-E183K/R/D represents ZmC2/WHP1 with E to K/R/D mutation at 183rd amino acid. **(A)** Western blot (anti-His-tag) after prokaryotic expression. 6xHis-tag was added at protein’s N terminal. Fused protein was purified using Ni-NTA column. T, *E. coli* cells after IPTG induction (total protein); P, precipitant of lysate of *E. coli* cells (precipitated protein); S, supernatant of lysate of *E. coli* cells (soluble protein); F, flow-through products from column; E, elution product from column (purified protein). **(B)** Transient expression of CHS in maize leaf protoplasts. GFP-tag was fused at protein’s C-terminal. **(C)** Stable expression of ZmC2 in leaf protoplasts of transgenic Arabidopsis. ZmC2-WT and ZmC2-E183K were fused with YFP-tag and transformed into *Arabidopsis tt4* mutant. For each gene, two independent transgenic lines (#1 and #2) were gained.

To evaluate the effect of the E183K mutation on protein structure or enzyme activity, ZmC2-WT and ZmC2-E183K were fused with His-tag to perform prokaryotic expression followed by protein extraction and purification. Western blot analysis showed that ZmC2-WT proteins exist in total protein, precipitated and soluble component after lysis and they could bind to and eventually be eluted from the Ni-NTA column. In contrast, ZmC2-E183K proteins could not be purified because they were almost completely precipitated/aggregated and barely existed in the supernatant after lysis (Figure 6A).

To further explore if ZmC2-E183K also aggregates in plant cells, the subcellular localization strategy was taken. The GFP (green fluorescent protein)-tag was fused at the C-terminus of ZmC2-WT and ZmC2-E183K, and the reconstructed plasmids were transferred into maize leaf protoplasts for transient expression. ZmC2-WT proteins existed as evenly distributed in the cytoplasm without aggregation (type I) or with some aggregation (type II). Notably, ZmC2-E183K fused proteins existed as totally aggregated (type III) (Figure 6B).

Another convincing evidence for the aggregation of ZmC2-E183K was gained through Arabidopsis transformation. ZmC2-WT and ZmC2-E183K were fused with YFP (yellow fluorescent protein)-tag at their C-terminus and stably expressed in *Arabidopsis tt4* mutant. ZmC2-WT fused proteins were evenly distributed in the cytoplasm (type I) in independent transgenic lines, while ZmC2-E183K significantly aggregated into some points (type III) (Figure 6C).

Similar phenomena were observed for ZmWHP1, the most identical protein of ZmC2. In prokaryotic expression, ZmWHP1-WT proteins were detected as soluble and precipitated, while ZmWHP1-E183K proteins completely aggregated/precipitated (Figure 6A); when transiently expressed in maize leaf protoplasts, ZmWHP1-WT showed evenly distributed or with some aggregation, while ZmWHP1-E183K totally aggregated (Figure 6B). Besides, additional point mutations were deployed on E183 of ZmC2, including ZmC2-E183R and ZmC2-E183D. E and D are both acidic amino acids with similar structures, while R and K are both basic amino acids. All mutant-type proteins showed almost completely aggregated in prokaryotic expression and transient expression in maize protoplasts (Figures 6A,B). As E and D are both acidic amino acids and their side-chain are -(CH_2_)_2_-COOH and -CH_2_-COOH respectively, the E183D mutation leading to protein aggregation may indicate a restrictive structural requirement, i.e., chain-length of the acidic side chain. In summary, E183K and other mutations on E183 could cause protein aggregation of chalcone synthase, leading to dysfunction.

### A Proposed Aggregation Model for ZmC2-E183K

**FIGURE 7:**
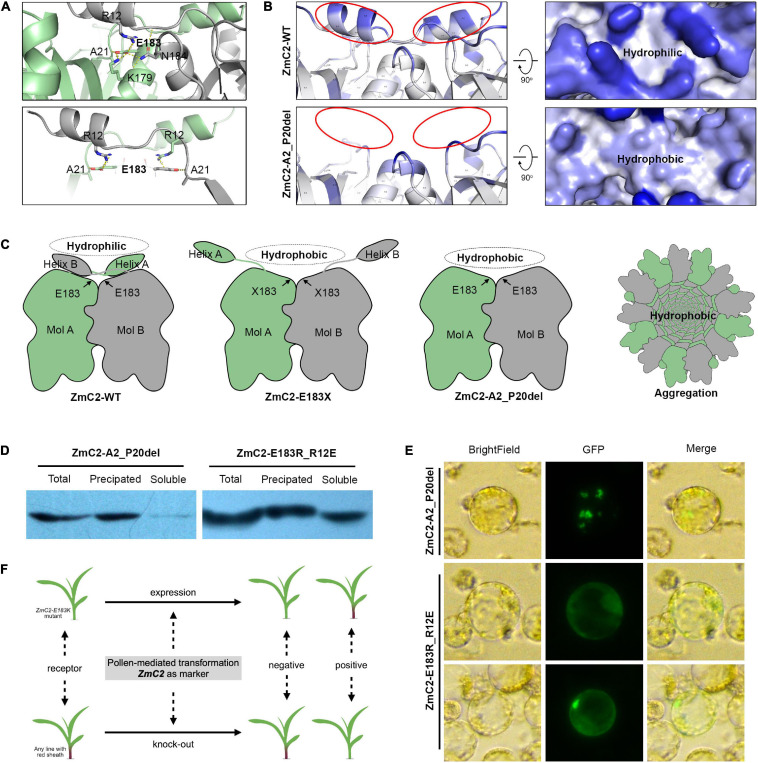
Proposed aggregation model. ZmC2-WT represents the wild-type chalcone synthase ZmC2; ZmC2-E183K, ZmC2 with E to K missense mutation at 183rd amino acid; ZmC2-E183X, any missense mutation at E183; ZmC2-A2_P20del, deletion mutation from A2 to P20 (deletion of N-terminal helix); ZmC2-E183R_R12E, E183R and R12E double mutations. **(A)** Polar contacts analysis of E183 in ZmC2. The bottom panel highlights the contacts between E183 and N-terminal helix. **(B)** Solvent accessible analysis of ZmC2-WT and ZmC2-A2_P20del. **(C)** Simplified protein structure and aggregation model. **(D)** Western blot after prokaryotic expression for ZmC2-A2_P20del and ZmC2-E183R_R12E. Proteins were fused with 6xHis-tag at N-terminal. **(E)** Transient expression of ZmC2-A2_P20del and ZmC2-E183R_R12E. Proteins were fused with GFP-tag at C-terminal. **(F)** Potential usage of *ZmC2* gene and *ZmC2-E183K* mutant line in maize transgene.

Structure modeling of ZmC2 indicated the wild-type proteins exist as homodimers, and the side chain of E183 has polar contacts with A21, K179 in the same molecule and R12, N184 in the paired molecule. Of them, A21 and R12 may be important for the conformation of N-terminal helix (Figure 7A). An aggregation model was proposed as following (Figures 7B,C). In wild-type chalcone synthase, the N-terminal helix could bend to cover the paired molecule, generating hydrophilic surface. Thus, the homodimer is stable to exist in cytoplasm or lysis. When mutation happens on E183, related polar contacts were broken; the N-terminal helix fails to cover the paired molecule and buried hydrophobic surface exposed. Thus, the dimer is not stable any more. The hydrophobic interactions drive proteins to aggregate and protect the hydrophobic surface inside.

According to this proposed model, the N-terminal helix is essential for generating stable dimer. We performed artifact damage on the N-terminal helix (ZmC2-A2_P20del, deletion mutation from A2 to P20). ZmC2-A2_P20del proteins were almost totally precipitated/aggregated in prokaryotic expression and transient expression in maize protoplasts (Figure 7D). As E183 has polar contacts with R12, we performed a complementary mutation (R12E) for ZmC2-E183R and achieved ZmC2-E183R_R12E with double mutations. From both prokaryotic expression and transient expression in maize protoplasts, we observed that the complementary mutation could at least partially rescue the aggregation of ZmC2-E183R (Figure 7E). All these indicate the importance of N-terminal helix and E183-R12 interaction for the stability of ZmC2 proteins.

## Discussion

### Consistence of Phenotype and the Protein Aggregation Model

The protein aggregation model is consistent with the observed phenotype (Figure 1). In B73 line or B73-type F2 progenies with rich color, there are only ZmC2-WT proteins, which are dimers and functional. In the mutant line or mutant-type F2 progenies which are colorless, there are only ZmC2-E183K proteins, which are aggregated and dysfunctional. According to “co-co assembly” mechanism (Bertolini et al., 2021), *ZmC2-WT* genes would generate ZmC2-WT homodimers directly, while *ZmC2-E183K* genes generate ZmC2-E183K aggregates directly, thus ZmC2-WT/ZmC2-E183K proteins (unknown status) only occupy a tiny proportion or even do not exist. In F1 or F1-type F2 progenies, there are half ZmC2-WT homodimers (functional) and half ZmC2-E183K aggregates (dysfunctional), which is completely consistent with the observed mid-colored phenotype. However, there may be other mechanisms for the aggregation instead of our proposed hydrophilic and/or hydrophobic model. Furthermore, *in vivo* experiments are in need to determine ZmC2-E183R, ZmC2-E183D, and ZmC2-E183R_R12E proteins are functional or not.

Protein aggregation raises strong interest in animal fields due to its involvement in human diseases (Clancy, 2008; Boeynaems et al., 2018; Guo et al., 2018). Previous knowledge in the plant field was limited to that stressful conditions could induce the aggregation of misfolded proteins (Nakajima and Suzuki, 2013; McLoughlin et al., 2019). Our work is the first to reveal the effect of genetic variations on protein aggregation in the plant field. However, additional characteristics of the mutation and protein aggregation are still under investigation.

### Comparison of *C2-E183K* and *C2-Idf* Mutants

In maize, previous studies on chalcone synthase focused on the *C2-Idf* mutant (Brink and Greenblatt, 1954). *C2-Idf* is a dominant mutant; homozygous *C2-Idf* plants and seeds do not show pigmentation, while heterozygotes show significantly reduced pigmentation (Dooner, 1983; Della Vedova et al., 2005). At the RNA level, *ZmC2* transcripts were significantly reduced to ∼20% in heterozygotes or nearly zero in homozygotes compared to the wild-type (Franken et al., 1991; Della Vedova et al., 2005). Della Vedova et al. (2005) demonstrated that there are three copies of *ZmC2* in *C2-Idf* mutant instead of a single copy, one of them may generate natural anti-sense transcript, leading to the dominant inhibitory effect through RNA silencing. Eloy et al. (2017) reported that *C2-Idf* mutants display strongly reduced levels of apigenin- and tricin-related flavonoids, resulting in impeded incorporation of tricin into the lignin polymer.

In this study, we identified the E183K mutation as a novel loss-of-function allele for *ZmC2*. Unlike *C2-Idf*, *C2-E183K* is a recessive mutant, heterozygotes show intermediate pigmentation level (Figure 1B). E183K has no effect on the transcript expression of *ZmC2* and other genes in related biosynthesis pathways (Figure 4D and Supplementary Figures 3–5). Instead, it causes protein aggregation of ZmC2, leading to dysfunction. Our metabolome results showed that in the first sheaths of *C2-E183K* mutants, the downstream flavonoids biosynthesis was blocked, while the metabolites in the biosynthesis of scopolin were up-regulated (Supplementary Figure 5). This novel loss-of-function mutant provides a new chance to investigate how CHS affects these biosynthesis pathways. As flavonoids are involved in multiple physiological processes, it is also of interest to explore the performance of the mutant under biotic and abiotic stresses in the future. Furthermore, a combination of the colorless mutant as acceptor material, *ZmC2* as a marker gene and the pollen mediated transformation may provide a simple and effective way for maize transgenic transformation and screening (Figure 7F).

### Advantage of Combining EMS Mutagenesis, MutMap Strategy, and Whole-Genome Sequencing

In this study, combining EMS mutagenesis, MutMap strategy and whole-genome sequencing, we identified the causal gene and mutation directly without further fine mapping. EMS mutagenesis has the advantage that most of the time, EMS induces C to T changes, resulting in C > T or G > A substitutions. The small size of SNPs or indels makes them easily detectable by current high-throughput sequencing technology. The other advantage of EMS mutagenesis is that multiple mutations are jointly and randomly distributed across the whole genome, which makes it possible to cover most genes and to establish as markers. A significant advantage of MutMap is the improvement of mapping population construction. In other methods, the mapping population is constructed by crossing two lines with distinct ecotypes. However, the high density of variations between the ecotypes makes it challenging to pinpoint the causal mutation. In MutMap, the mutant is crossed to the wild-type with the same ecotype, and variants induced by mutagenesis are taken as markers. Although density is relatively low, the markers are still sufficient for association and linkage analysis. Superior to RNA-Seq or exon-capture sequencing, whole-genome sequencing has the power to cover those variants exist in intergenic or regulatory regions. In our study, the colorless mutant (B73 ecotype) was crossed with B73. Only 5654 variants homozygous and unique in the mutant were taken as markers, and over 90% of them are C > T or G > A substitution (Figure 2 and Supplementary Figure 1). The EMS mutagenesis, MutMap strategy and whole-genome sequencing fully utilize the advantages of each other, and their combinations will accelerate and facilitate the process for forward genetics.

## Data Availability Statement

The raw data for whole genome sequencing are available at NCBI-SRA (PRJNA593329). Raw RNA-seq data are available at NCBI-SRA (PRJNA593349). Other relevant data are within the article and Supplementary Material. The datasets presented in this study can be found in online repositories. The names of the repository/repositories and accession number(s) can be found in the article/Supplementary Material.

## Author Contributions

YY, HD, and HL planned and designed the research. HD, HL, YX, SS, SL, XS, HKL, NJ, XW, and ZZ performed the experiments, analyzed the data, conducted the field work, etc. HD, HL, and YY wrote the manuscript. All authors contributed to the article and approved the submitted version.

## Conflict of Interest

The authors declare that the research was conducted in the absence of any commercial or financial relationships that could be construed as a potential conflict of interest.

## Footnotes

1 http://plants.ensembl.org

2 http://broadinstitute.github.io/picard/

3 https://nucleobytes.com/4peaks/index.html

4 https://www.ebi.ac.uk/Tools/psa/

5 https://swissmodel.expasy.org

6 https://plants.ensembl.org/biomart/martview/

7 www.shimadzu.com.cn/

8 www.appliedbiosystems.com.cn/

9 http://www.kegg.jp

10 https://blast.ncbi.nlm.nih.gov/

11 https://www.ebi.ac.uk/Tools/msa/clustalo/

12 https://www.ebi.ac.uk/Tools/msa/mview/

13 http://plants.ensembl.org/

14 https://www.ebi.ac.uk/gxa/home

## Supplementary Material

Supplementary Figure 1 Summary of detected variants by whole-genome sequencing.

Supplementary Figure 2 Multiple alignment of ZmC2, ZmWHP1, OsCHS1, and AtCHS.

Supplementary Figure 3 ZmC2-E183K mutation caused transcriptomic and metabolic changes in phenylpropanoid biosynthesis (kegg-zma00940) in maize first sheaths.

Supplementary Figure 4 ZmC2-E183K caused transcriptomic and metabolic changes in anthocyanin biosynthesis (kegg-zma00942) in maize first sheaths.

Supplementary Figure 5 ZmC2-E183K caused transcriptomic and metabolic changes in flavone and flavonol biosynthesis (kegg-zma00944) in maize first sheaths.

Supplementary Table 1 Statistics of whole-genome sequencing data for mutant mapping.

Supplementary Table 2 Information of markers for mutant mapping in Chr4:125M-250M (excel file).

Supplementary Table 3 Statistics of RNA-seq data.

Supplementary Table 4 Differentially expressed genes detected by RNA-Seq (excel file).

Supplementary Table 5 Differentially expressed metabolites detected by widely targeted metabolome (excel file).

Supplementary Table 6 Information of ZmC2 and its 1581 homologs (excel files).

Supplementary Table 7 Primers used in this study.

## References

[B1] Abe A. Kosugi S. Yoshida K. Natsume S. Takagi H. (2012). Genome sequencing reveals agronomically important loci in rice using MutMap. *Nat. Biotechnol.* 30 174–178. 10.1038/nbt.2095 22267009

[B2] Agati G. Azzarello E. Pollastri S. Tattini M. (2012). Flavonoids as antioxidants in plants: location and functional significance. *Plant Sci.* 196 67–76. 10.1016/j.plantsci.2012.07.014 23017900

[B3] An J.-P. Liu X. Li H.-H. You C.-X. Wang X.-F. (2017). Apple RING E3 ligase MdMIEL1 inhibits anthocyanin accumulation by ubiquitinating and degrading MdMYB1 protein. *Plant Cell Physiol.* 58 1953–1962. 10.1093/pcp/pcx129 29016961

[B4] Austin R. S. Vidaurre D. Stamatiou G. Breit R. Provart N. J. (2011). Next-generation mapping of Arabidopsis genes. *Plant J.* 67 715–725. 10.1111/j.1365-313x.2011.04619.x 21518053

[B5] Bechtold N. Ellis J. Pelletier G. (1993). In planta Agrobacterium mediated gene transfer by infiltration of adult Arabidopsis thaliana plants. *C R Acad. Sci. Paris Life Sci.* 316 1194–1199.

[B6] Bertolini M. Fenzl K. Kats I. Wruck F. Tippmann F. (2021). Interactions between nascent proteins translated by adjacent ribosomes drive homomer assembly. *Science* 371 57–64. 10.1126/science.abc7151 33384371 PMC7613021

[B7] Bieza K. Lois R. (2001). An Arabidopsis mutant tolerant to lethal ultraviolet-B levels shows constitutively elevated accumulation of flavonoids and other phenolics. *Plant Physiol.* 126 1105–1115. 10.1104/pp.126.3.1105 11457961 PMC116467

[B8] Boeynaems S. Alberti S. Fawzi N. L. Mittag T. Polymenidou M. (2018). Protein Phase Separation: A New Phase in Cell Biology. *Trends Cell Biol.* 28 420–435. 10.1016/j.tcb.2018.02.004 29602697 PMC6034118

[B9] Bolger A. M. Lohse M. Usadel B. (2014). Trimmomatic: a flexible trimmer for Illumina sequence data. *Bioinformatics* 30 2114–2120. 10.1093/bioinformatics/btu170 24695404 PMC4103590

[B10] Brink R. A. Greenblatt I. M. (1954). Diffuse, a pattern gene in maize. *J. Hered* 45 47–50. 10.1093/oxfordjournals.jhered.a106436

[B11] Chen W. Gong L. Guo Z. (2013). A Novel Integrated Method for Large-Scale Detection, Identification, and Quantification of Widely Targeted Metabolites: Application in the Study of Rice Metabolomics. *Mol. Plant* 6 1769–1780. 10.1093/mp/sst080 23702596

[B12] Chong J. Xia J. (2018). MetaboAnalystR: an R package for flexible and reproducible analysis of metabolomics data. *Bioinformatics* 34 4313–4314. 10.1093/bioinformatics/bty528 29955821 PMC6289126

[B13] Cingolani P. Platts A. Wang L. L. Coon M. Nguyen T. (2012). A program for annotating and predicting the effects of single nucleotide polymorphisms, SnpEff: SNPs in the genome of Drosophila melanogaster strain w1118; iso-2; iso-3. *Fly* 6 80–92. 10.4161/fly.19695 22728672 PMC3679285

[B14] Clancy S. (2008). Genetic mutation. *Nat. Educat.* 1:187.

[B15] Cuperus J. T. Montgomery T. A. Fahlgren N. Burke R. T. Townsend T. (2010). Identification of MIR390a precursor processing-defective mutants in Arabidopsis by direct genome sequencing. *Proc. Natl. Acad. Sci. U. S. A.* 107 466–471. 10.1073/pnas.0913203107 20018656 PMC2806713

[B16] Della Vedova C. B. Lorbiecke R. Kirsch H. Schulte M. B. Scheets K. (2005). The dominant inhibitory chalcone synthase allele C2-Idf (inhibitor diffuse) from Zea mays (L.) acts via an endogenous RNA silencing mechanism. *Genetics* 170 1989–2002. 10.1534/genetics.105.043406 15956664 PMC1449766

[B17] Dooner H. K. (1983). Coordinate genetic regulation of flavonoid biosynthetic enzymes in maize. *Mol. General Genet.* 189 136–141. 10.1007/bf00326066

[B18] Eloy N. B. Voorend W. Lan W. Saleme M. de L. S. Cesarino I. (2017). Silencing CHALCONE SYNTHASE in Maize Impedes the Incorporation of Tricin into Lignin and Increases Lignin Content. *Plant Physiol.* 173 998–1016. 10.1104/pp.16.01108 27940492 PMC5291018

[B19] Falcone Ferreyra M. L. Rius S. P. Casati P. (2012). Flavonoids: biosynthesis, biological functions, and biotechnological applications. *Front. Plant Sci.* 3:222. 10.3389/fpls.2012.00222 23060891 PMC3460232

[B20] Feller A. Machemer K. Braun E. L. Grotewold E. (2011). Evolutionary and comparative analysis of MYB and bHLH plant transcription factors. *Plant J.* 66 94–116. 10.1111/j.1365-313x.2010.04459.x 21443626

[B21] Franken P. Niesbach-Klosgen U. Weydemann U. Marechal-Drouard L. Saedler H. (1991). The duplicated chalcone synthase genes C2 and Whp (white pollen) of Zea mays are independently regulated; evidence for translational control of Whp expression by the anthocyanin intensifying gene in. *EMBO J.* 10 2605–2612. 10.1002/j.1460-2075.1991.tb07802.x 1714383 PMC452959

[B22] Ghosh D. Konishi T. (2007). Anthocyanins and anthocyanin-rich extracts: role in diabetes and eye function. *Asia Pac. J. Clin. Nutr.* 16 200–208. 17468073

[B23] Gonzalez A. Zhao M. Leavitt J. M. Lloyd A. M. (2008). Regulation of the anthocyanin biosynthetic pathway by the TTG1/bHLH/Myb transcriptional complex in Arabidopsis seedlings. *Plant J.* 53 814–827. 10.1111/j.1365-313x.2007.03373.x 18036197

[B24] Guo F. Liu X. Cai H. Le W. (2018). Autophagy in neurodegenerative diseases: pathogenesis and therapy. *Brain Pathol.* 28 3–13. 10.1111/bpa.12545 28703923 PMC5739982

[B25] He Z. Zhang H. Gao S. Lercher M. J. Chen W. (2016). Evolview v2: an online visualization and management tool for customized and annotated phylogenetic trees. *Nucleic Acids Res.* 44 W236–W241. 27131786 10.1093/nar/gkw370 PMC4987921

[B26] Hill J. T. Demarest B. L. Bisgrove B. W. Gorsi B. Su Y.-C. (2013). MMAPPR: mutation mapping analysis pipeline for pooled RNA-seq. *Genome Res.* 23 687–697. 10.1101/gr.146936.112 23299975 PMC3613585

[B27] Ho S. N. Hunt H. D. Horton R. M. Pullen J. K. Pease L. R. (1989). Site-directed mutagenesis by overlap extension using the polymerase chain reaction. *Gene* 77 51–59. 10.1016/0378-1119(89)90358-2 2744487

[B28] Holton T. A. Cornish E. C. (1995). Genetics and Biochemistry of Anthocyanin Biosynthesis. *Plant Cell* 7 1071–1083. 10.2307/3870058 12242398 PMC160913

[B29] Jeon J. S. Lee S. Jung K. H. Jun S. H. Jeong D. H. (2000). T-DNA insertional mutagenesis for functional genomics in rice. *Plant J.* 22 561–570. 10886776 10.1046/j.1365-313x.2000.00767.x

[B30] Jia S. Li A. Morton K. Avoles-Kianian P. Kianian S. F. (2016). A Population of Deletion Mutants and an Integrated Mapping and Exome-seq Pipeline for Gene Discovery in Maize. *G3* 6 2385–2395. 10.1534/g3.116.030528 27261000 PMC4978893

[B31] Jiao Y. Peluso P. Shi J. Liang T. Stitzer M. C. (2017). Improved maize reference genome with single-molecule technologies. *Nature* 546 524–527. 28605751 10.1038/nature22971 PMC7052699

[B32] Jin H. Cominelli E. Bailey P. Parr A. Mehrtens F. (2000). Transcriptional repression by AtMYB4 controls production of UV-protecting sunscreens in Arabidopsis. *EMBO J.* 19 6150–6161. 10.1093/emboj/19.22.6150 11080161 PMC305818

[B33] Kanehisa M. Goto S. (2000). KEGG: kyoto encyclopedia of genes and genomes. *Nucleic Acids Res.* 28 27–30. 10592173 10.1093/nar/28.1.27 PMC102409

[B34] Kim D. Langmead B. Salzberg S. L. (2015). HISAT: a fast spliced aligner with low memory requirements. *Nat. Methods* 12 357–360. 10.1038/nmeth.3317 25751142 PMC4655817

[B35] Koornneef M. (1990). Mutations affecting the testa color in Arabidopsis. *Arabidop. Inf. Serv.* 28 1–4.

[B36] Krysan P. J. Young J. C. Sussman M. R. (1999). T-DNA as an insertional mutagen in Arabidopsis. *Plant Cell* 11 2283–2290. 10.2307/3870955 10590158 PMC144136

[B37] Lee H. S. Wicker L. (1991). Anthocyanin Pigments in the Skin of Lychee Fruit. *J. Food Sci.* 56 466–468. 10.1111/j.1365-2621.1991.tb05305.x

[B38] Li H. Durbin R. (2009). Fast and accurate short read alignment with Burrows-Wheeler transform. *Bioinformatics* 25 1754–1760. 10.1093/bioinformatics/btp324 19451168 PMC2705234

[B39] Li H. Handsaker B. Wysoker A. Fennell T. Ruan J. (2009). The Sequence Alignment/Map format and SAMtools. *Bioinformatics* 25 2078–2079. 10.1093/bioinformatics/btp352 19505943 PMC2723002

[B40] Li J. Ou-Lee T. M. Raba R. Amundson R. G. Last R. L. (1993). Arabidopsis Flavonoid Mutants Are Hypersensitive to UV-B Irradiation. *Plant Cell* 5 171–179. 10.2307/3869583 12271060 PMC160260

[B41] Li P. Li Y.-J. Zhang F.-J. Zhang G.-Z. Jiang X.-Y. (2017). The Arabidopsis UDP-glycosyltransferases UGT79B2 and UGT79B3, contribute to cold, salt and drought stress tolerance via modulating anthocyanin accumulation. *Plant J.* 89 85–103. 10.1111/tpj.13324 27599367

[B42] Lin B.-W. Gong C.-C. Song H.-F. Cui Y.-Y. (2017). Effects of anthocyanins on the prevention and treatment of cancer. *Br. J. Pharmacol.* 174 1226–1243. 10.1111/bph.13627 27646173 PMC5429338

[B43] Lin Y. Zhang C. Lan H. Gao S. Liu H. (2014). Validation of Potential Reference Genes for qPCR in Maize across Abiotic Stresses, Hormone Treatments, and Tissue Types. *PLoS One* 9:e95445. 10.1371/journal.pone.0095445 24810581 PMC4014480

[B44] Liu S. Yeh C.-T. Tang H. M. Nettleton D. Schnable P. S. (2012). Gene mapping via bulked segregant RNA-Seq (BSR-Seq). *PLoS One* 7:e36406. 10.1371/journal.pone.0036406 22586469 PMC3346754

[B45] Livak K. J. Schmittgen T. D. (2001). Analysis of relative gene expression data using real-time quantitative PCR and the 2(-Delta Delta C(T)) Method. *Methods* 25 402–408. 10.1006/meth.2001.1262 11846609

[B46] Love M. I. Huber W. Anders S. (2014). Moderated estimation of fold change and dispersion for RNA-seq data with DESeq2. *Genome Biol.* 15:550. 25516281 10.1186/s13059-014-0550-8 PMC4302049

[B47] Lu X. Liu J. Ren W. Yang Q. Chai Z. (2018). Gene-Indexed Mutations in Maize. *Mol. Plant* 11 496–504. 10.1016/j.molp.2017.11.013 29223623

[B48] Luo W. Brouwer C. (2013). Pathview: an R/Bioconductor package for pathway-based data integration and visualization. *Bioinformatics* 29 1830–1831. 10.1093/bioinformatics/btt285 23740750 PMC3702256

[B49] Madeira F. Park Y. M. Lee J. Buso N. Gur T. (2019). The EMBL-EBI search and sequence analysis tools APIs in 2019. *Nucleic Acids Res.* 47 W636–W641. 30976793 10.1093/nar/gkz268 PMC6602479

[B50] Magwene P. M. Willis J. H. Kelly J. K. (2011). The statistics of bulk segregant analysis using next generation sequencing. *PLoS Comput. Biol.* 7:e1002255. 10.1371/journal.pcbi.1002255 22072954 PMC3207950

[B51] McCarty D. R. Settles A. M. Suzuki M. Tan B. C. Latshaw S. (2005). Steady-state transposon mutagenesis in inbred maize. *Plant J.* 44 52–61. 10.1111/j.1365-313x.2005.02509.x 16167895

[B52] McKenna A. Hanna M. Banks E. Sivachenko A. Cibulskis K. Kernytsky A. (2010). The Genome Analysis Toolkit: a MapReduce framework for analyzing next-generation DNA sequencing data. *Genome Res.* 20 1297–1303. 10.1101/gr.107524.110 20644199 PMC2928508

[B53] McLoughlin F. Kim M. Marshall R. S. Vierstra R. D. Vierling E. (2019). HSP101 Interacts with the Proteasome and Promotes the Clearance of Ubiquitylated Protein Aggregates. *Plant Physiol.* 180 1829–1847. 10.1104/pp.19.00263 31113833 PMC6670096

[B54] Michelmore R. W. Paran I. Kesseli R. V. (1991). Identification of markers linked to disease-resistance genes by bulked segregant analysis: a rapid method to detect markers in specific genomic regions by using segregating populations. *Proc. Natl. Acad. Sci. U. S. A.* 88 9828–9832. 10.1073/pnas.88.21.9828 1682921 PMC52814

[B55] Nakajima Y. Suzuki S. (2013). Environmental stresses induce misfolded protein aggregation in plant cells in a microtubule-dependent manner. *Int. J. Mol. Sci.* 14 7771–7783. 10.3390/ijms14047771 23574938 PMC3645715

[B56] Papatheodorou I. Moreno P. Jonathan M. Fuentes A. M.-P. George N. (2020). Expression Atlas update: from tissues to single cells. *Nucleic Acids Res.* 48 D77–D83. 31665515 10.1093/nar/gkz947 PMC7145605

[B57] Patel R. K. Jain M. (2012). NGS QC Toolkit: a toolkit for quality control of next generation sequencing data. *PLoS One* 7:e30619. 10.1371/journal.pone.0030619 22312429 PMC3270013

[B58] R Core Team,(2019). *R: A Language and Environment for Statistical Computing.* Austria: R Foundation for Statistical Computing.

[B59] Ren M. Wang Z. Xue M. Wang X. Zhang F. (2019). Constitutive expression of an A-5 subgroup member in the DREB transcription factor subfamily from Ammopiptanthus mongolicus enhanced abiotic stress tolerance and anthocyanin accumulation in transgenic Arabidopsis. *PLoS One* 14:e0224296. 10.1371/journal.pone.0224296 31644601 PMC6808444

[B60] Schaefer H. M. Schaefer V. Levey D. J. (2004). How plant-animal interactions signal new insights in communication. *Trends Ecol. Evol.* 19 577–584. 10.1016/j.tree.2004.08.003

[B61] Schmelzer E. Jahnen W. Hahlbrock K. (1988). In situ localization of light-induced chalcone synthase mRNA, chalcone synthase, and flavonoid end products in epidermal cells of parsley leaves. *Proc. Natl. Acad. Sci. U. S. A.* 85 2989–2993. 10.1073/pnas.85.9.2989 16578833 PMC280128

[B62] Schneeberger K. Ossowski S. Lanz C. Juul T. Petersen A. H. (2009). SHOREmap: simultaneous mapping and mutation identification by deep sequencing. *Nat. Methods* 6 550–551. 10.1038/nmeth0809-550 19644454

[B63] Schrodinger LLC, (2015). *The PyMOL Molecular Graphics System, Version 2.1.*

[B64] Strygina K. V. Kochetov A. V. Khlestkina E. K. (2019). Genetic control of anthocyanin pigmentation of potato tissues. *BMC Genet.* 20:27. 10.1186/s12863-019-0728-x 30885125 PMC6421638

[B65] Sun C. Deng L. Du M. Zhao J. Chen Q. (2020). A Transcriptional Network Promotes Anthocyanin Biosynthesis in Tomato Flesh. *Mol. Plant* 13 42–58. 10.1016/j.molp.2019.10.010 31678614

[B66] Takagi H. Tamiru M. Abe A. Yoshida K. Uemura A. (2015). MutMap accelerates breeding of a salt-tolerant rice cultivar. *Nat. Biotechnol.* 33 445–449. 10.1038/nbt.3188 25798936

[B67] Tran Q. H. Bui N. H. Kappel C. Dau N. T. N. Nguyen L. T. (2020). Mapping-by-Sequencing via MutMap Identifies a Mutation in ZmCLE7 Underlying Fasciation in a Newly Developed EMS Mutant Population in an Elite Tropical Maize Inbred. *Genes* 11:281. 10.3390/genes11030281 32155750 PMC7140824

[B68] Tyedmers J. Mogk A. Bukau B. (2010). Cellular strategies for controlling protein aggregation. *Nat. Rev. Mol. Cell Biol.* 11 777–788. 10.1038/nrm2993 20944667

[B69] Wallace T. C. Slavin M. Frankenfeld C. L. (2016). Systematic Review of Anthocyanins and Markers of Cardiovascular Disease. *Nutrients* 8:32. 10.3390/nu8010032 26761031 PMC4728646

[B70] Walley J. W. Sartor R. C. Shen Z. Schmitz R. J. Wu K. J. (2016). Integration of omic networks in a developmental atlas of maize. *Science* 353 814–818. 10.1126/science.aag1125 27540173 PMC5808982

[B71] Waterhouse A. Bertoni M. Bienert S. Studer G. Tauriello G. (2018). SWISS-MODEL: homology modelling of protein structures and complexes. *Nucleic Acids Res.* 46 W296–W303. 29788355 10.1093/nar/gky427 PMC6030848

[B72] Williams C. A. Grayer R. J. (2004). Anthocyanins and other flavonoids. *Nat. Product. Rep.* 21 539–573. 10.1039/b311404j 15282635

[B73] Winkel-Shirley B. (2001). Flavonoid biosynthesis. A colorful model for genetics, biochemistry, cell biology, and biotechnology. *Plant Physiol.* 126 485–493. 10.1104/pp.126.2.485 11402179 PMC1540115

[B74] Xu W. Dubos C. Lepiniec L. (2015). Transcriptional control of flavonoid biosynthesis by MYB-bHLH-WDR complexes. *Trends Plant Sci.* 20 176–185. 10.1016/j.tplants.2014.12.001 25577424

[B75] Zhai Z. Jung H.-I. Vatamaniuk O. K. (2009). Isolation of protoplasts from tissues of 14-day-old seedlings of Arabidopsis thaliana. *J. Vsualiz. Exp.* 30:1149. 10.3791/1149 19687783 PMC3142859

