# Supplementary material for: E183K Mutation in Chalcone Synthase C2 Causes Protein Aggregation and Maize Colorless
Source: Front Plant Sci. 2021 Jun 23;12:679654. doi: 10.3389/fpls.2021.679654 (PMC8261305; doi:10.3389/fpls.2021.679654)
Supplement: Supplementary Figure 1 — Summary of detected variants by whole-genome sequencing. [file Data_Sheet_1.docx]

**E183K mutation in chalcone synthase C2 causes protein aggregation and maize colorless**

Haixiao Dong^1#^, He Li^1#^, Yingjie Xue^1^, Shengzhong Su^1^, Shipeng Li^1^, Xiaohui Shan^1^, Hongkui Liu^1^, Nan Jiang^1^, Xuyang Wu^1^, Zhiwu Zhang^2^, Yaping Yuan^1^*

^1^College of Plant Science, Jilin University, Changchun, China 130062

^2^Department of Crop and Soil Sciences, Washington State University, Pullman, USA 99164

^*^corresponding to YY (yuanyp@jlu.edu.cn)

^#^These authors contribute equally

**Supplementary figures
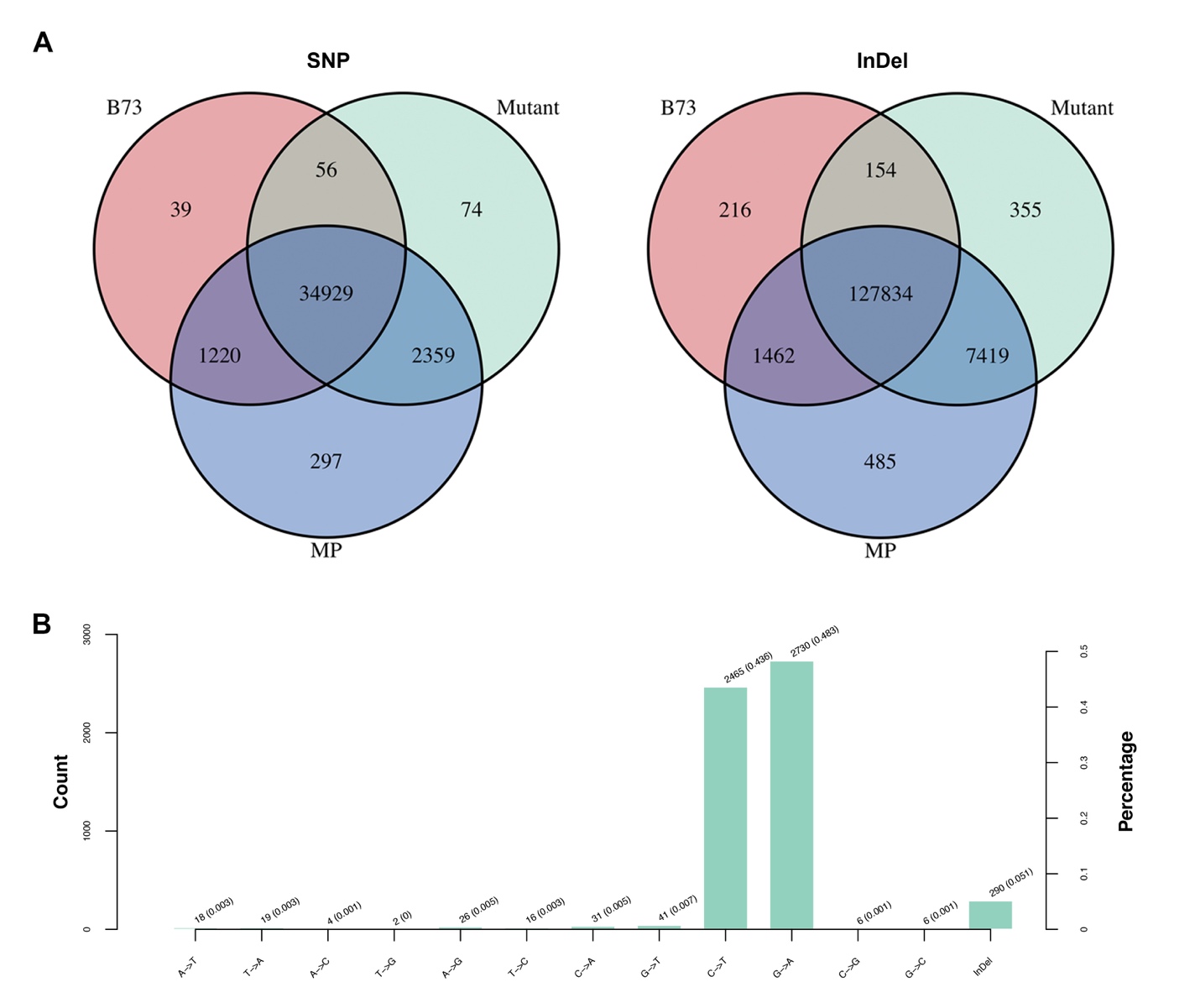
**

**Figure S1.** **Summary of detected variants by whole-genome sequencing**

**(A)** Venn plot for all detected variants. MP, pool of mutant-type F2 progenies.

**(B)** Summary on the type of 5,654 markers used for mutant mapping.

**Figure S2. Multiple alignment of ZmC2, ZmWHP1, OsCHS1 and AtCHS.**

ZmC2, chalcone synthase C2 [*Zea mays*] (NCBI accession: NP_001142246.1); ZmWHP1, chalcone synthase WHP1 [*Zea mays*] (NCBI accession: NP_001149022.1); OsCHS1, chalcone synthase 1 [*Oryza sativa Japonica Group*] (NCBI accession: XP_015618054.1); AtCHS, Chalcone and stilbene synthase family protein [*Arabidopsis thaliana*] (NCBI accession: NP_196897.1).

**
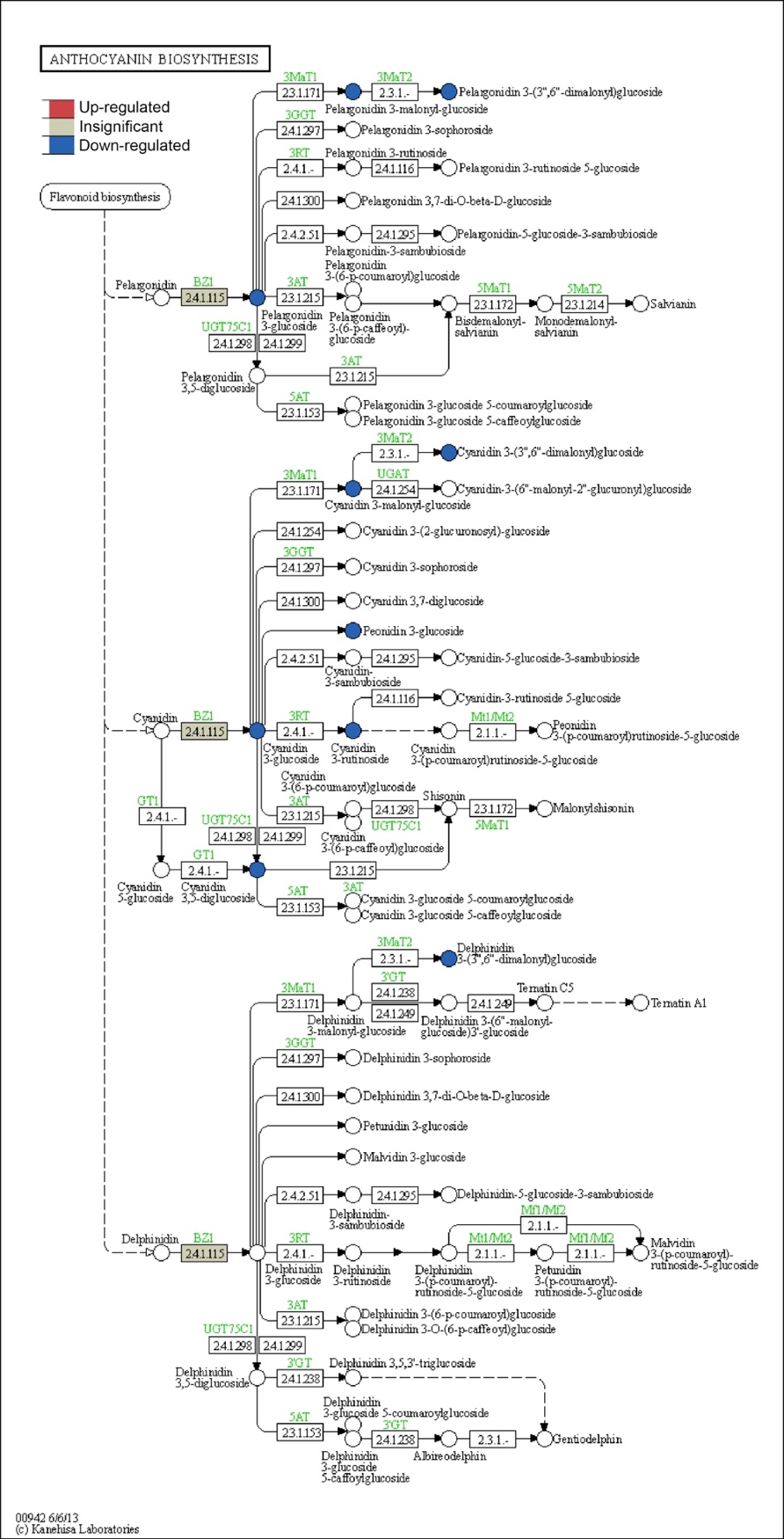
**

**Figure S3. ZmC2-E183K mutation caused transcriptomic and metabolic changes in anthocyanin biosynthesis (kegg-zma00942) in maize first sheaths**


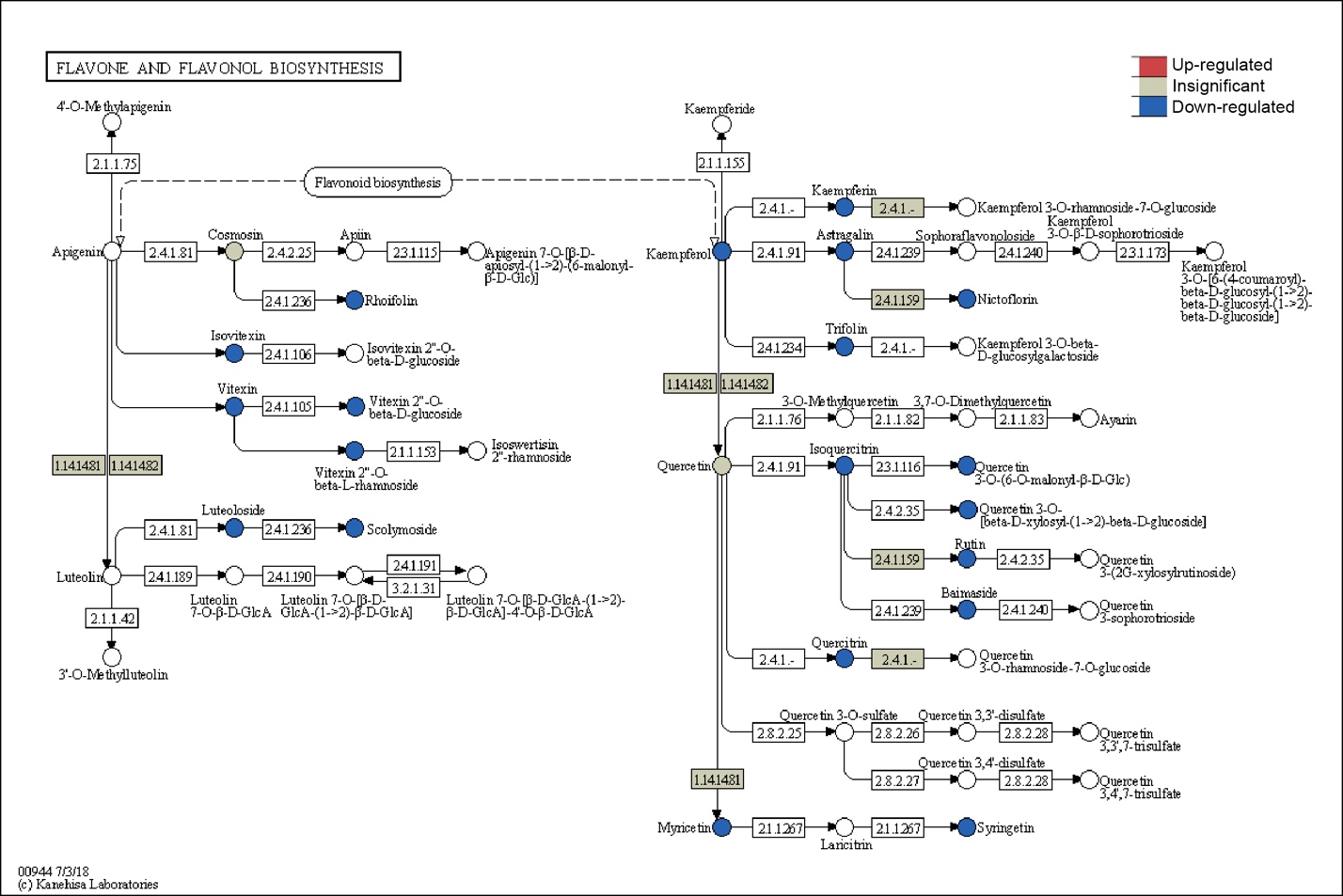


**Figure S4. ZmC2-E183K mutation caused transcriptomic and metabolic changes in flavone and flavonol biosynthesis (kegg-zma00944) in maize first sheaths.**


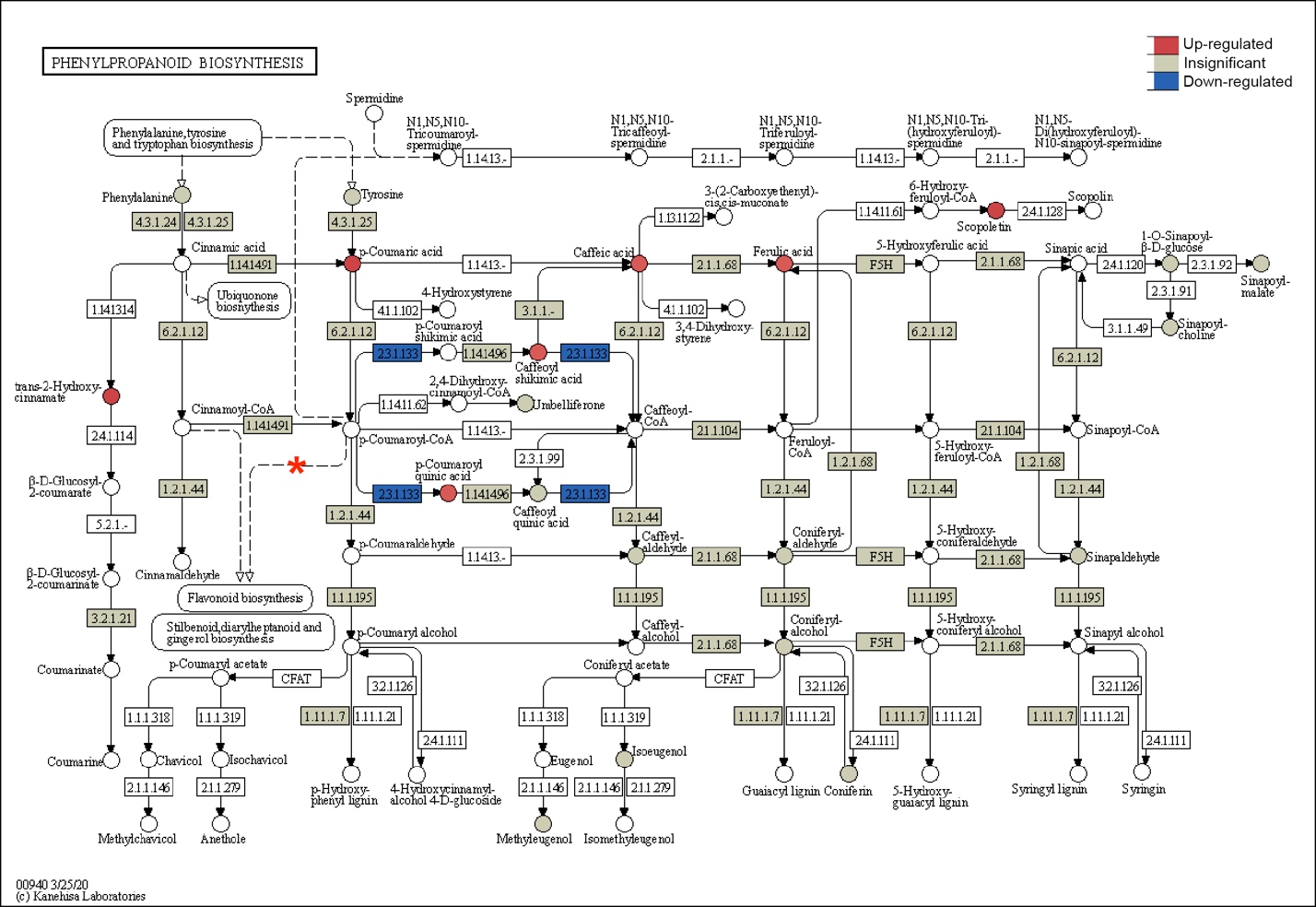


**Figure S5. ZmC2-E183K mutation caused transcriptomic and metabolic changes in phenylpropanoid biosynthesis (kegg-zma00940) in maize first sheaths.**

The red asterisk labels ZmC2 involved reaction.

**Supplementary tables**

**Table S1.** Statistics of whole-genome sequencing data for mutant mapping.

| **Sample** | **Clean_reads** | **Mapped rate (%)** | **Average_depth** | **Cov_ratio_1X (%)** | **Cov_ratio_5X (%)** | **Cov_ratio_10X (%)** |
| --- | --- | --- | --- | --- | --- | --- |
| B73 | 232,275,938 | 99.62 | 14.15 | 99.14 | 96.04 | 76.57 |
| Mutant | 332,771,740 | 96.17 | 19.43 | 99.29 | 97.87 | 91.10 |
| MP | 1,179,584,256 | 99.49 | 61.70 | 99.36 | 98.77 | 98.01 |

**Note:** MP is a pool of mutant-type F2 progeny.

**Table S2.** Information of markers for mutant mapping in Chr4:125M-250M (excel file).

**Table S3.** Statistics of RNA-seq data.

|  | **MP1** | **MP2** | **BP1** | **BP2** |
| --- | --- | --- | --- | --- |
| Total reads | 26381014(100.00%) | 28132094(100.00%) | 27949800(100.00%) | 31200696(100.00%) |
| Total mapped | 25812767(97.85%) | 27554904(97.95%) | 27297018(97.66%) | 30529864(97.85%) |
| Mutiple mapped | 3092641(11.72%) | 3782524(13.45%) | 4023551(14.40%) | 2866981(9.19%) |
| Uniquely mapped | 22720126(86.12%) | 23772380(84.50%) | 23273467(83.27%) | 27662883(88.66%) |
| Read-1 mapped | 11374654(43.12%) | 11897768(42.29%) | 11643268(41.66%) | 13848147(44.38%) |
| Read-2 mapped | 11345472(43.01%) | 11874612(42.21%) | 11630199(41.61%) | 13814736(44.28%) |
| Reads map to '+' | 11363850(43.08%) | 11888575(42.26%) | 11650410(41.68%) | 13837111(44.35%) |
| Reads map to '-' | 11356276(43.05%) | 11883805(42.24%) | 11623057(41.59%) | 13825772(44.31%) |
| Non-splice reads | 16378300(62.08%) | 16828232(59.82%) | 16559459(59.25%) | 19303132(61.87%) |
| Splice reads | 6341826(24.04%) | 6944148(24.68%) | 6714008(24.02%) | 8359751(26.79%) |
| Reads mapped in proper pairs | 21913744(83.07%) | 22951382(81.58%) | 22392656(80.12%) | 26743812(85.72%) |

**Note:** BP1 and BP2 are the two biological replicates of pool of B73-type F2 progeny; MP1 and MP2 are pools of mutant-type F2 progeny.

**Table S4.** Differentially expressed genes detected by RNA-Seq (excel).

**Table S5.** Differentially expressed metabolites detected by widely-targeted metabolome (excel).

**Table S6.** Information of ZmC2 and its 1861 homologs (excel).

**Table S7.** Primers used in this study.

| **Primer**  **ID** | **Primer/Product Name** | **Forward Primer** | **Reverse Primer** | **Target**  **Gene** | **Template** | **Product**  **Size (bp)** | **Assay and PCR program** | **Purpose** |
| --- | --- | --- | --- | --- | --- | --- | --- | --- |
| #1 | ZmC2-SangerValidation | GGTACGGTTTGTTTTAATTGGGC | CGAGTCGGGCAGGATGGTC | Surrounding region of *ZmC2*-E183 site | Corresponding maize DNA | 620 | 2xEs Taq MasterMix (Dye) | Sanger sequencing validation of *ZmC2*-E183 site |
| #2 | ZmC2-RT | ATGATCCGGAAGCGTTACATGCA | CGAGTCGGGCAGGATGGT | *ZmC2-WT*, *ZmC2-E183K* | Corresponding Arabidopsis cDNA | 561 | 2xEs Taq MasterMix (Dye) | RT-PCR of *ZmC2* in *Arabidopsis* |
| #3 | AtACT2-RT | AGTCTTGTTCCAGCCCTCGT | GAGATCCACATCTGCTGGAATG | *AtACT2* | Corresponding Arabidopsis cDNA | 297 | 2xEs Taq MasterMix (Dye) | Internal control for RT-PCR in *Arabidopsis* |
| #4 | ZmC2-WT/E183K-precursor | CTGCTGTCGCGTGGTAGACG | TAGCTCTACCCTGGTCTTGCAT | *ZmC2-WT*, *ZmC2-E183K* | Corresponding maize cDNA | 1741 | PrimeSTAR HS DNA polymerase | Cloning a longer precursor containing *ZmC2-WT* or *ZmC2-E183K* |
| #5 | ZmWHP1-WT-precursor | GAAAGAGCTCCTCCGACGAC | GTCGTTCATGTCTGGAGCGA | *ZmWHP1-WT* | cDNA of maize B73 line | 1342 | PrimeSTAR HS DNA polymerase | Cloning a longer precursor containing *ZmWHP1-WT*. |
| #6-1 | ZmC2-E183R-fragment1 | GCGTGGCCAAGGACCTCGCGaggAACAACCGCGGCGCGCGGGT | sames as ZmC2-WT-precursor-Reverse | *ZmC2-E183R* | ZmC2-WT-precursor | 907 | PrimeSTAR HS DNA polymerase | Introduce artifact E183R mutation through overlap PCR during precursor cloning. |
| #6-2 | ZmC2-E183R-fragment2 | same as ZmC2-WT-precursor-Forward | ACCCGCGCGCCGCGGTTGTTcctCGCGAGGTCCTTGGCCACGC |  | ZmC2-WT-precursor | 671 | PrimeSTAR HS DNA polymerase |  |
| #6-3 | ZmC2-E183R-precursor | same as ZmC2-WT-precursor-Forward | same as ZmC2-WT-precursor-Reverse |  | ZmC2-E183R-fragment1 + ZmC2-E183R-fragment2 | 1741 | PrimeSTAR HS DNA polymerase |  |
| #7-1 | ZmC2-E183D-fragment1 | GCGTGGCCAAGGACCTCGCGgacAACAACCGCGGCGCGCGGGT | same as ZmC2-WT-precursor-Reverse | *ZmC2-E183D* | ZmC2-WT-precursor | 907 | PrimeSTAR HS DNA polymerase | Introduce artifact E183D mutation through overlap PCR during precursor cloning. |
| #7-2 | ZmC2-E183D-fragment2 | same as ZmC2-WT-precursor-Forward  Reverse: | ACCCGCGCGCCGCGGTTGTTgtcCGCGAGGTCCTTGGCCACGC |  | ZmC2-WT-precursor | 671 | PrimeSTAR HS DNA polymerase |  |
| #7-3 | ZmC2-E183D-precursor | same as ZmC2WT-precursor-Forward | same as ZmC2WT-precursor-Reverse |  | ZmC2-E183D-fragment1 + ZmC2-E183D-fragment2 | 1741 | PrimeSTAR HS DNA polymerase |  |
| #8-1 | ZmWHP1-E183K-fragment1 | GCGTGGCCAAGGACCTGGCGaagAACAACCGCGGGGCGAGGGT | same as ZmWHP1-precursor | *ZmWHP1-E183K* | ZmWHP1-WT-precursor | 721 | PrimeSTAR HS DNA polymerase | Introduce artifact E183K mutation through overlap PCR during precursor cloning. |
| #8-2 | ZmWHP1-E183K-fragment2 | same as ZmWHP1-WT-precursor-Forward | ACCCTCGCCCCGCGGTTGTTcttCGCCAGGTCCTTGGCCACGC |  | ZmWHP1-WT-precursor | 664 | PrimeSTAR HS DNA polymerase |  |
| #8-3 | ZmWHP1-E183K-precursor | same as ZmWHP1-WT-precursor-Forward | same as ZmWHP1-WT-precursor-Reverse |  | ZmWHP1-E183K-fragment1 + ZmWHP1-E183K-fragment2 | 1342 | PrimeSTAR HS DNA polymerase |  |
| #9 | ZmC2-prokaryotic | catATGGCCGGCGCGACCGTGA | ctcgagGGCGGTGGCCGCTCCG | *ZmC2-WT*, *ZmC2-E183K*, *ZmC2-E183R*,  *ZmC2-E183D* | Corresponding precursor | 1209 | PrimeSTAR HS DNA polymerase | Construct vector for prokaryotic expression |
| #10 | ZmC2-E183R_R12E-prokaryotic | catATGGCCGGCGCGACCGTGACCGTGGAGGAGGTGgagAAGGCCCAGCGCGCCACCG | same as ZmC2-prokaryotic-Reverse | *ZmC2-E183R_R12E* | ZmC2-E183R-precursor | 1209 | PrimeSTAR HS DNA polymerase | Construct vector prokaryotic expression |
| #11 | ZmC2-A2_P20del-prokaryotic | catatgGCCACCGTGCTGGCGATCG | same as ZmC2-prokaryotic-Reverse. | *ZmC2-A2_P20del* | ZmC2-WT-precursor | 1152 | PrimeSTAR HS DNA polymerase | Construct vector for prokaryotic expression |
| #12 | ZmWHP1-prokaryotic | catATGGCCGGCGCCACCGTGAC | ctcgagGGCGGCGGTGGGCGCTC | *ZmWHP1-WT*, *ZmWHP1-E183K* | Corresponding precursors | 1212 | PrimeSTAR HS DNA polymerase | Construct vector for prokaryotic expression |
| #13 | ZmC2-plant | ggggACAAgTTTgTACAAAAAAgCAggCTTCATGGCCGGCGCGACCGTGA | ggggACCACTTTgTACAAgAAAgCTgggTTGGCGGTGGCCGCTCCGGTG | *ZmC2-WT*, *ZmC2-E183K*, *ZmC2-E183R*, *ZmC2-E183D*, *ZmC2-E183R_R12E* | Corresponding precursors or prokaryotic expression vectors | 1261 | PrimeSTAR HS DNA polymerase | Construct vector for transient expression in maize leaf protoplasts and stable expression in Arabidopsis. |
| #14 | ZmC2-A2_P20del-plant | ggggACAAgTTTgTACAAAAAAgCAggCTTCATGGCCACCGTGCTGGCGATCG | same as ZmC2-transgene-Reverse | *ZmC2-A2_P20del* | Corresponding prokaryotic expression vector | 1204 | PrimeSTAR HS DNA polymerase | Construct vector for transient expression in maize leaf protoplasts. |
| #15 | ZmWHP1-plant | ggggACAAgTTTgTACAAAAAAgCAggCTTCATGGCCGGCGCCACCGTGAC | ggggACCACTTTgTACAAgAAAgCTgggTTGGCGGCGGTGGGCGCTC | *ZmWHP1-WT*, *ZmWHP1-E183K* | Corresponding prokaryotic expression vectors | 1264 | PrimeSTAR HS DNA polymerase | Construct vector for transient expression in maize leaf protoplast |

**Note:** The underlined letters are restriction sites or gateway adaptors. For construction of prokaryotic expression vectors, *Nde* I (catatg) and *Xho* I (ctcgag) enzyme cutting sites were used. For construction of plant expression vectors, gateway systems were used. For gene cloning in need of high-fidelity, PrimeSTAR HS DNA Polymerase (Order No. R010, Takara, Beijing, China) was used. The PCR programs were: 30 cycles of 98℃ 10 sec, 55℃ 5 sec, 72℃ 1 min/kb. When high-fidelity not required, 2xEs Taq MaterMix (Dye) (Order No. CW0690, CWBIO, Beijing, China) was used. The PCR programs were: step1, 94℃ 2min; step2, 35 cycles of 94℃ 30 sec, 55℃ 30 sec, 72℃ 30 sec/kb; step 3, 72℃ 2min.
